# Exploring IDP–Ligand Interactions: Tau K18 as a Test Case

**DOI:** 10.3390/ijms21155257

**Published:** 2020-07-24

**Authors:** Darius Vagrys, James Davidson, Ijen Chen, Roderick E. Hubbard, Ben Davis

**Affiliations:** 1 Vernalis Research, Granta Park, Great Abington, Cambridge CB21 6GB, UK; darius.vagrys@gmail.com (D.V.); j.davidson@vernalis.com (J.D.); i.chen@vernalis.com (I.C.); r.hubbard@vernalis.com (R.E.H.); 2 YSBL, Department of Chemistry, University of York, Heslington, York YO10 5DD, UK

**Keywords:** intrinsically disordered protein, tau K18, nuclear magnetic resonance, surface plasmon resonance, microscale thermophoresis

## Abstract

Over the past decade intrinsically disordered proteins (IDPs) have emerged as a biologically important class of proteins, many of which are of therapeutic relevance. Here, we investigated the interactions between a model IDP system, tau K18, and nine literature compounds that have been reported as having an effect on tau in order to identify a robust IDP–ligand system for the optimization of a range of biophysical methods. We used NMR, surface plasmon resonance (SPR) and microscale thermophoresis (MST) methods to investigate the binding of these compounds to tau K18; only one showed unambiguous interaction with tau K18. Several near neighbors of this compound were synthesized and their interactions with tau K18 characterized using additional NMR methods, including 1D ligand-observed NMR, diffusion-ordered spectroscopy (DOSY) and ^19^F NMR. This study demonstrates that it is possible to detect and characterize IDP–ligand interactions using biophysical methods. However, care must be taken to account for possible artefacts, particularly the impact of compound solubility and where the protein has to be immobilized.

## 1. Introduction

The classical view of proteins has closely related function to a stable three-dimensional (3D) structure and the paradigm of “Sequence → Structure → Function” has dominated scientific thinking since early in the 20th century [1,2]. By the time the first experimentally derived 3D models of protein structure were available, this “Structure → Function” relationship was widely accepted and underpins our understanding and appreciation of most protein structures [3,4]. It was in 1975 that the first ideas of peptides and proteins (glucagon), having a biological function without having a defined structure, emerged [5]. Since then, multiple proteins have been identified to be either completely disordered, or have disordered regions [6,7].

The importance of the biological role and function of partially or fully disordered proteins has grown in recent years, leading to an increasing interest in discovery of compounds to affect the behaviour of IDPs in a potentially therapeutic manner. However, the identification and characterization of IDP–ligand interactions has been challenging [8,9,10].

The aggregation-prone tau protein has been identified to be disordered and is involved in multiple tauopathies, including Pick’s disease, frontotemporal dementia Parkinsonism linked to chromosome 17 (FTDP-17), progressive supranuclear palsy and Alzheimer’s disease [11,12,13,14]. With an increasingly aging population, the number of people affected by Alzheimer’s disorder will increase, and together with it, the therapeutic relevance of dementia-related targets, such as tau protein [15]. Tau aggregates, found in brain tissue, exhibit increased levels of phosphorylation and mostly contain double helical stacks, or paired helical filaments (PHFs) [16]. It has also been demonstrated that two hexapeptide motifs PHF6(VQIVYK) and PHF6*(VQIINK) play an important role in the formation of PHFs and are found at the core of formed fibrils [17,18,19]. These motifs are located in imperfect repeat regions R, namely in 2R and 3R, of the microtubule-binding domain. In addition to this, the formation of fibrils appears to be enhanced by oxidation of Cys residues and interactions of tau with polyanionic molecules in vitro, such as heparin [20,21].

To date, a range of molecules have been identified as modulators of the tau aggregation process and state, including polyphenols, rhodanines, benzothiazoles, and phenothiazines, typically using thioflavin-dye based assays with limited biophysical characterization [22,23,24,25,26,27,28,29,30,31,32]. Because of the importance of PHF6 and PHF6* regions in fibril formation, tau K18 and tau K19 constructs, containing 4 or 3 repeat regions, respectively, have been used extensively as model systems in order to identify and evaluate low molecular weight inhibitors of aggregation [19]. However, the interactions between tau and these ligands have not been extensively characterized with widely used biophysical methods.

Here, we have used a range of biophysical techniques in order to characterize and explore the interactions between monomeric tau K18 and several literature ligands. The experience gained through these studies, in particular an understanding of possible sources for artefacts, will help in the application of the repertoire of biophysical methods to study IDP–ligand interactions. The development and optimisation of these methods may help to enable drug discovery campaigns against IDPs.

## 2. Results

### 2.1. Protein Production

Tau K18 forms aggregates in solution over time, associated with intermolecular cysteine coupling [20]. Mutation of C291 and C322 to serine (C291S, C322S) is known to increase stability, while it is claimed that the mutant protein maintains similar interactions. A construct for tau K18 (C291S, C322S), hereafter tau K18^M^, was expressed and purified as previously described [33]. The biotinylation of AviTag-tau K18^M^ using BirA enzyme and incorporation of 3-fluoro Tyr as Y310 to tau K18^M^ were achieved following previously published methods [34,35].

### 2.2. NMR Identifies Cl-NQTrp to Interact with Monomeric tau K18^M^

**Figure 1:**
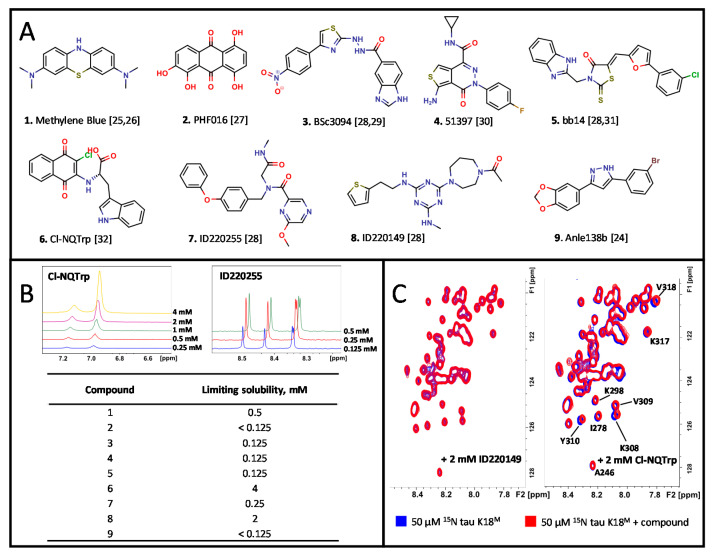
Literature ligand NMR titration assay against tau K18^M^. (**A**) Chemical structures of the ligands (CPK colouring scheme). (**B**) An example of 1D ^1^H NMR spectra during titration of Cl-NQTrp and ID220255 as determined by 1D ^1^H NMR with determined ligand solubilities. (**C**) The 2D [^1^H-^15^N] SF-HMQC spectra of a non-binder ID220149 (2 mM) (Left) and a binder Cl-NQTrp (2 mM) (Right).

The aqueous solubility of nine tau ligands were evaluated with 1D ^1^H NMR by measuring the integral of a specific peak for the ligand while increasing its concentration to the point where the signal failed to increase linearly (Figure 1A, B). The soluble ligands were then screened against monomeric ^15^N-labeled tau K18^M^ using 2D SF-HMQC pulse sequence which produces spectra that are used to detect changes to the backbone amide signals (Figure 1C, Figure S1) [36]. It was determined that only compound 6 (Cl-NQTrp) produced significant chemical shift perturbations (CSPs), mainly affecting the PHF6 region. The 2D [^1^H-^13^C] HSQC spectrum indicated that the easily distinguishable side chains of aromatic residues of tau K18^M^ were also affected by compound **6** (Y310 and F346) (Figure S2). CSPs were defined as significant if the shift was more than half of a peak width and dose-dependent. 

### 2.3. SPR and MST Identifies Additional Compounds to Interact with tau K18^M^

**Figure 2:**
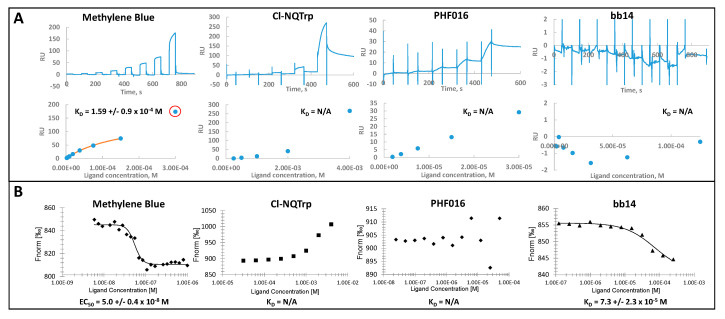
Surface plasmon resonance (SPR) and microscale thermophoresis (MST) techniques were used to assess ligand binding to tau K18^M^. (**A**) Kinetic SPR binding curves and steady-state affinity graphs for Methylene Blue, Cl-NQTrp, PHF016 and bb14 with calculated K_D_ values. Red-circled data point marks excluded data. Immobilization level: 1000 RU of biotinylated AviTag-tau K18^M^. (**B**) Methylene Blue, Cl-NQTrp, PHF016 and bb14 MST dose–response data with assessed K_D_ or EC_50_ values to tau K18^M^.

Biotinylated AviTag-tau K18^M^ was immobilized on a streptavidin-derivatized dextran surface for SPR experiments. A dose–response titration assay identified Methylene Blue, PHF016, and Cl-NQTrp as interacting (Figure 2A, Figure S3). ID220149 exhibited lower than theoretical R_max_ (~23 RU) responses and was considered to be a potential artefact (Figure S3). The Methylene Blue SPR kinetic curves indicated potential non-specific interactions with the immobilized protein on the surface at higher concentrations (>150 µM). The partially saturating steady-state binding profile was used to calculate K_D_ value of 159 +/− 9 µM. PHF016 showed slow-on, slow-off behaviour with linear dose–response curve with no saturation above determined theoretical R_max_, indicating non-stoichiometric interactions. Cl-NQTrp, (shown to interact with tau K18^M^ by NMR), interacted with the protein in non-stoichiometric manner at higher concentrations (>1 mM). bb14 did not interact with tau K18 to any detectable extent.

The overlap of the spectrum of intrinsic fluorescence of Methylene Blue and RED-Tris-NTA dye at λ = 600–650 nm prevented the direct labeling of the protein with the dye for MST experiments. However, a label-free approach was possible. Tau K18^M^ was titrated from 5.9 to 2000 nM onto 100 nM of Methylene Blue and an EC_50_ of 50 +/− 4 nM was determined with Hill coefficient of 4 (Figure 2B). The data could not be fitted to a standard 1:1 model suggesting that multiple molecules may interact with the protein at the same time.

For other MST experiments, tau K18^M^ was labeled with RED-Tris-NTA dye. The MST data for Cl-NQTrp suggested that the ligand interacted with tau K18^M^ in a dose-dependent manner, confirming the results reported above with NMR and SPR (Figure 2B, Figure S4). The affinity of Cl-NQTrp could not be reliably determined because the MST data did not reach saturation. While compound 2 (PHF016) data could not be fitted to a binding model, compound 5 (bb14) was identified to interact with the labeled protein with a K_D_ of 73 +/− 23 µM (1:1 binding model).

### 2.4. Detailed Characterization of Cl-NQTrp and Its Interactions with tau K18^M^

Cl-NQTrp contains electrophilic quinone and chloro-enone groups; HPLC-MS and NMR experiments confirmed that the compound does not form covalent adducts with tau K18^M^ or dithiothreitol (DTT) (Figure S5). The IDP–ligand interactions were also determined to be not non-specific or solely electrostatic in nature, as CSPs remained of comparable magnitude at increasing NaCl concentrations, and no interactions were observed with a fragment of Cl-NQTrp, L-Trp (Figure S6, Figure S7).

**Figure 3:**
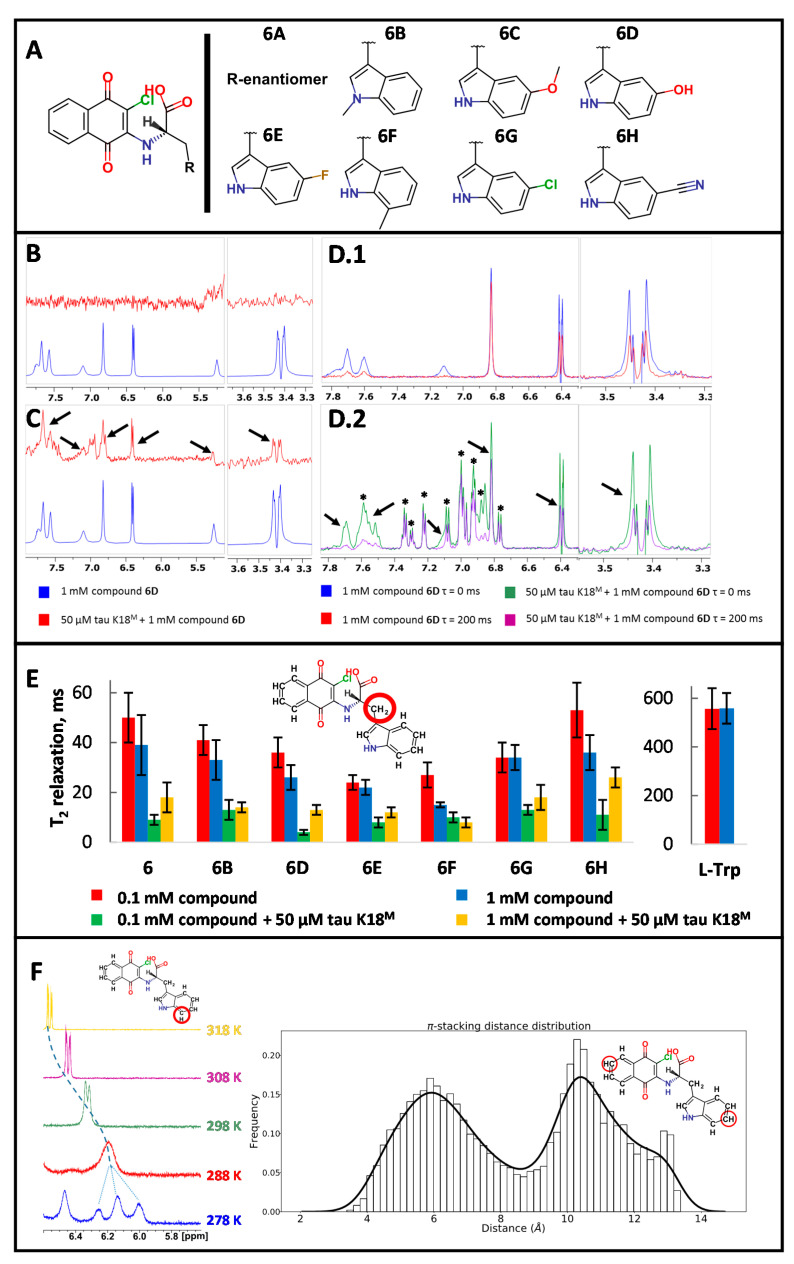
1D ligand-observed NMR studies with Cl-NQTrp and its near neighbors (NNs). (**A**) Synthesized NNs of Cl-NQTrp. (**B**) Saturation transfer difference (STD) data (red) indicates no ligand-specific signals when compared to ^1^H reference spectrum (blue) for compound 6D. (**C**) Water–ligand observed via gradient spectroscopy (water-LOGSY) data (red) indicates ligand-specific peaks (black arrows) when compared to a ^1^H reference spectrum (blue) for compound 6D. (**D**) ^1^H Carr–Purcell–Meiboom–Gill (CPMG)-filtered data for compound 6D in the absence (**D.1**) and presence (**D.2**) of tau K18^M^ (protein-derived peaks marked as **∗**) indicates no IDP–ligand interactions using standard (200ms) relaxation delays as ligand-specific peaks (black arrows) decreased in intensity at a similar level after CPMG filter. (**E**) Comparison of T_2_ relaxation times for tryptophan methylene group (red circled) at different concentrations of compound **6** and its NNs in the presence or absence of tau K18^M^. L-Trp T_2_ relaxation times were measured without any protein present. (**F**) ^1^H NMR spectral changes for red-circled proton of Cl-NQTrp indicating decreasing intensity, coalescence, splitting, and sharpening of the signal with decreasing temperature indicating intermediate chemical exchange events (Left). Over a 1 µs simulation in explicit water, Cl-NQTrp adopts either folded or unfolded conformation close to 50% of the time (Right). Atom pair distance: 4 to 8 Å between the red-circled carbon atoms in folded and 9–13 Å in unfolded conformations.

To further explore the SAR for compound binding, eight near-neighbors (NNs) of Cl-NQTrp were synthesized (Figure 3A). All NNs were observed to produce similar CSPs in 2D [^1^H-^15^N] SF-HMQC spectra (Figure S8). Interestingly, tau K18^M^ formed a precipitate at higher concentrations of NNs (>2 mM), particularly with 6E and 6G (>1 mM) (Figure S9). This effect was not due to limiting compound solubility.

Three widely used ligand-observed NMR methods were used to assess Cl-NQTrp interactions with tau K18^M^. These are the saturation transfer difference (STD) experiment which utilizes direct magnetization transfer from protein to bound ligand [37], the water–ligand observed via gradient spectroscopy (water-LOGSY) experiment [38] which detects phase-sensitive NOE transfer from water molecules to both free and bound ligands, and a T_2_ relaxation-filtered experiment which identifies small molecules which show rapid T_2_ relaxation [39]; this rapid T_2_ relaxation (short T_2_ relaxation time) is typically the result of interactions between the ligand and a slowly-tumbling protein.

No signal indicative of binding was observed in STD data acquired for the tau K18^M^–Cl-NQTrp complex (Figure 3B). Water-LOGSY data indicated that Cl-NQTrp interacts with tau K18^M^, particularly for compound 6D (Figure 3C). Interestingly, compound 6D exhibited a short T_2_ relaxation time using standard relaxation delays in the ^1^H T_2_-filtered experiment even when the tau K18^M^ sample was not present (Figure 3D). However, this rapid T_2_ relaxation was both unchanged over a wide range of ligand concentrations and also in the presence of 0.2% Tween-20, indicating that the T_2_ relaxation observed is due to intramolecular rather than intermolecular interactions (Figure 3E, Figure S10, Table S2).

In order to account for the observed short T_2_ relaxation times and to evaluate T_2_ relaxation for Cl-NQTrp and its NNs more accurately, extensive ^1^H Carr–Purcell–Meiboom–Gill (CPMG)-filtered experiments were performed in the absence and presence of tau K18^M^. The readily assigned tryptophan methylene protons were used to track the changes in T_2_ relaxation time of each molecule under different conditions (Figure 3E, Table S2). While the T_2_ values were comparable for Cl-NQTrp and its NNs at 0.1 and 1 mM without protein present, the values decreased in the presence of tau K18^M^, consistent with an interaction between the ligand and IDP, as the rapidly tumbling ligand moves more slowly upon binding to a slowly-tumbling protein. Such data outcome agrees with our previous observations by 2D [^1^H-^15^N] SF-HMQC and water-LOGSY (Figure 1C, Figure 3C). Additionally, T_2_ relaxation times for the methylene protons of L-Trp were measured as being one order of magnitude longer than for Cl-NQTrp, indicating that short T_2_ values observed for Cl-NQTrp are compound-specific (Figure 3E, Table S2).

Temperature gradient experiments were performed from 278 K to 318 K for Cl-NQTrp. These indicated intermediate chemical exchange events which may contribute to the observed short T_2_ relaxation times observed earlier (Figure 3D,E,F). The exchange events can be observed as ligand-specific peaks in the 1D ^1^H spectrum decrease in intensity, coalesce, split, and sharpen again with decreasing temperature. This may result in part from exchange between stacked and non-stacked indole:napthoquinone conformations, which causes a significant change in the local chemical environment on the NMR time scale as predicted by molecular dynamics simulation (Figure 3F).

**Figure 4:**
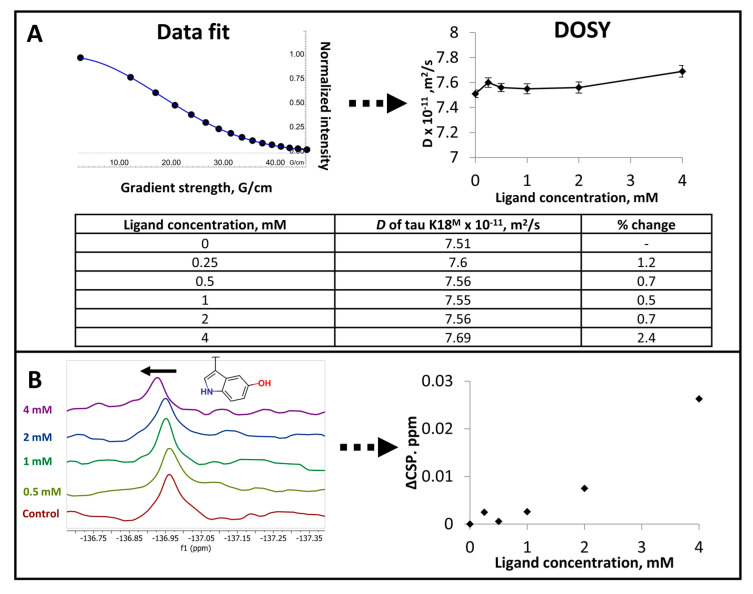
Investigating tau K18^M^ and Cl-NQTrp interactions using diffusion-ordered spectroscopy (DOSY) and ^19^F NMR. (**A**) The determination of diffusion rate *D* for 50 µM tau K18^M^ in the presence of different concentrations of compound 6C. (**B**) ^19^F NMR spectra for 50 µM tau K18^M^ (3-fluoro-Y310) and the observed chemical shift perturbations (CSPs) in the presence of compound 6D. Black arrow denote the vector direction of CSPs.

It was hypothesized that Cl-NQTrp and its NNs may also affect the diffusion rate *D* of tau K18^M^ at concentrations below those which resulted in precipitation. Diffusion ordered spectroscopy (DOSY) measures *D* value for individual NMR resonances, identifying any changes to the diffusion rate of the ligand as it interacts with the target protein, and vice versa [40,41]. DOSY data indicated a small but potentially significant change in *D* value for tau K18^M^, particularly in the presence of compound 6C (Figure 4A).

The ^19^F nucleus is highly sensitive to its local chemical environment [42]. Since one of the affected amino acid residues was Y310, as indicated by 2D [^1^H-^15^N] SF-HMQC and [^1^H-^13^C] HSQC spectra (Figure 1, Figure S1, Figure S2), ^19^F NMR was used to probe the IDP–ligand interactions using fluorine-labeled tau K18^M^ (3-fluoro-Y310) and detect CSPs caused by the ligand. However, ^19^F NMR data showed only small CSPs in the presence of compound 6D with a non-saturating dose response profile (Figure 4B).

## 3. Discussion

Recent research has identified a number of therapeutically relevant intrinsically disordered proteins (IDPs), leading to growing interest in identifying small molecules which affect their function [6,7]. However, due to the disordered nature of IDPs, it is necessary to establish and optimize reliable experimental protocols for biophysical methods to characterize IDP–ligand interactions. In order to do this, we have studied the interaction of a model tau system with a range of available ligands from literature studies using a suite of widely used biophysical techniques.

Tau protein is an IDP whose aggregation has been linked to multiple tauopathies [11,12,13,14]. While many tau aggregation inhibitors have been identified, there is limited experimental evidence for these compounds interacting with tau [22,23,24,25,26,27,28,29,30,31,32] and no broad studies applying a consistent set of methods. Methylene Blue and BSc3094 have been shown previously to interact with native tau K18 using 2D HSQC and 1D STD NMR methods, mapping the perturbed backbone amides and ligand protons, respectively.

It has been reported that capping or mutating Cys residues inhibits the aggregation of wild-type tau K18 [43]. In order to improve the stability of the protein over time during lengthy NMR experiments, we mutated Cys to Ser to increase the stability of tau K18^M^ while maintaining similar intramolecular and intermolecular interactions.

Typically, 1D NMR screening methods, such as STD, wLOGSY and CPMG-filtered, are used as a primary screening step in order to identify and prioritize binders from a compound library [37,38,39,44]. Following the identification of potent ligands, 2D [^1^H-^15^N] HSQC NMR can then be used to map a potential binding site. If the compound is of sufficient solubility, 2D HSQC NMR is considered to be a “gold standard” for detecting and characterizing protein–ligand interactions as the spectra identify specific backbone amides of the protein that are perturbed on ligand binding. The 2D SOFAST-HMQC (SF-HMQC) experiment is a rapid alternative to the conventional HSQC technique, and is particularly well suited for screening interactions with IDPs due to lack of dispersion in the amide region of the IDP spectrum compared to a typical folded protein [36].

Since this study was designed to robustly identify a model IDP–ligand system, and then to investigate the application of biophysical methods to this system, the “gold standard” 2D SF-HMQC experiment was used as a primary screen in order to identify a validated intermolecular interaction. Further, close near-neighbors of the ligand identified by 2D SF-HMQC were synthesized in order to ensure that the observed perturbations were not simply artefacts resulting from a contaminant in the original screening sample as has been reported previously [45].

The nine tau ligands screened are all commercially available and have been reported to modulate tau activity [24,25,26,27,28,29,30,31,32]. The titration experiments were performed in the presence of monomeric form of tau K18^M^. Only Cl-NQTrp appeared to bind robustly to this model IDP (Figure 1 and Figure S1). Other ligands may interact with the oligomeric rather than monomeric form of tau K18, as suggested previously [23]; such compounds would not have been identified by this screening cascade, which was designed solely to identify a ligand for monomeric tau K18. The compounds ID220255 and ID220149, for example, have been detected to bind to wild-type full length tau protein via high-throughput chemical microarray SPR (HT-CM-SPR) imaging screen and to also inhibit tau aggregation [28]. The same ligands were also identified to inhibit the aggregation of wild-type tau K18, indicating that the interactions between ligand and protein are occurring in the wild-type tau K18 part of the protein. As Methylene Blue has been shown to oxidize Cys residues of tau protein, the C291S and C322S mutations present in the tau K18 construct used here may have impaired the binding of Methylene Blue compared to previous NMR studies [25].

While Methylene Blue, bb14, and PHF016 were identified to interact with tau K18^M^ by MST or SPR, but not by 2D [^1^H-^15^N] SF-HMQC, it is possible that such data are not representative due to immobilization of the IDP on the dextran surface or the use of dye-labeling (Figure 2). Any conformational restrictions of an IDP may cause the protein to assume a physiologically irrelevant conformation, causing artefactual results. The SPR angle shifts and the observed responses have been previously shown to be sensitive to changes of pH, ionic strength and conformation of the immobilized protein [46,47]. While immobilized folded proteins can maintain their rigidity and interact with ligands, the structure of IDPs is likely to be perturbed upon interaction with a ligand due to their dynamic structure.

Methylene Blue did not show any apparent interaction with tau K18^M^ in 2D [^1^H-^15^N] SF-HMQC NMR spectra (Figure 1), whereas an interaction was identified by MST (K_D_ 50 +/− 4 nM) (Figure 2). Previously, it has been reported that Methylene Blue interacts with wild type tau K18(C291,C322) with a K_D_ of 125.8 +/− 5.4 nM, as determined by MST [48], which is broadly consistent with the value determined in our study. Previous investigations of wild type tau K18 have also suggested that the Cys residues may be involved in binding Methylene Blue [25,48]. However, our MST results using mutated tau K18 (tau K18^M^), which lacks these Cys residues, showed a similar interaction suggesting that the Cys residues are not critical. However, it is possible that non-specific or indirect interactions between the compound and dye-labeled tau may give rise to artefactual results in MST, particularly since in our studies no interaction is observed by NMR.

Electron-rich quinone and chloro-enone chemical groups, as are present in Cl-NQTrp, belong to the pan-assay interference (PAINS) group of moieties which can give rise to false positives in a wide range of assays, specifically by interfering with colorimetric readout or by forming covalent adducts with proteins [49]. The MS study showed no indication of covalently bound ligand to the protein, ruling out covalent modification of tau K18^M^ as a mode-of-action (Figure S5).

Limited SAR has shown that NNs produce similar CSPs to the parent molecule Cl-NQTrp when interacting with tau K18^M^ (Figure 3A, Figure S8). Of the 1D ligand-observed NMR methods, only ^1^H water-LOGSY and ^1^H CPMG-filtered experiments detected the binding of Cl-NQTrp and NNs to tau K18^M^; this is particularly evident for compound 6D (Figure 3C,E). The STD experiment relies upon saturation of nuclei in the hydrophobic core of a protein; this saturation spreads across the protein via through-space coupling, and is subsequently transferred to a bound ligand in the same manner. The lack of STD signal for binding of Cl-NQTrp to tau K18^M^ may simply be due to the lack of an intact hydrophobic core in the protein [37], but may also result from the lack of persistent protein structure through which magnetization can be transferred to reach any ligand binding site. In contrast, water-LOGSY, which does report an interaction between Cl-NQTrp and tau K18^M^, does not require the presence of a folded structure since magnetization is transferred from water molecules to the ligand via protein–ligand or protein–ligand–water complexes [38]. 

Initial CPMG-filtered experiments using standard T_2_ relaxation delays did not indicate any Cl-NQTrp–tau K18^M^ interactions (Figure 3(D.1,D.2)). However, this was found to potentially be due to the unusually short T_2_ relaxation time of Cl-NQTrp itself. Therefore, shorter T_2_ delays were used to further probe protein–ligand interactions and the acquired data was consistent with binding (Figure 3C). The observed short T_2_ relaxation times were attributed to the intramolecular chemical exchange events rather than aggregate formation, since the T_2_ relaxation time did not change upon addition of a detergent and the T_2_ relaxation time is invariant with ligand concentration [39,50]. The chemical exchange appears to occur when Cl-NQTrp molecule changes between “open” and “closed” conformations of the indole and napthoquinone groups as identified through molecular dynamics (Figure 3F).

The diffusion rate *D* of tau K18^M^ is affected slightly (<5%), particularly in the presence of compound 6C (Figure 4A). It is not clear, however, whether this change is significant and indeed whether this change results directly from a ligand binding event. Previous investigation of bovine pancreatic trypsin inhibitor (BPTI) has shown that the 58 amino acid long fully unfolded peptide has 13 to 20% lower diffusion rate than partially folded peptide [41]. Interestingly, compounds 6E and 6G cause tau K18^M^ to precipitate out of solution at >1 mM, which is not related to limiting compound solubility (Figure S9). The precipitation phenomenon did not cause any observable change in diffusion rate *D* and 2D [^1^H-^15^N] SF-HMQC spectra. As NMR DOSY can only measure diffusion of soluble components, any diffusion changes for insoluble components cannot be evaluated. It is possible that the structural changes caused by ligand binding would need to be both large enough and to not cause precipitation of the target protein in order to observe changes in diffusion rate. In addition to this, such change would potentially be observable in 2D [^1^H-^15^N] SF-HMQC spectra as the system becomes more ordered. It would be of interest to further investigate diffusion changes using more precise methods.

As ^19^F NMR chemical shifts are sensitive to the local environment [42], specific fluorine labeling was used to probe tau K18^M^–Cl-NQTrp interactions. The chemical shifts of tau K18^M^ Y310 were perturbed by compound 6D in 2D [^1^H-^15^N] SF-HMQC spectra. Similar perturbations were also observed for Y(3-fluoro)310 in ^19^F NMR spectra, but these were of relatively small magnitude (Figure 4B). This is somewhat surprising given the sensitivity of the ^19^F nucleus. However, prediction of ^19^F chemical shift perturbation remains challenging [51] and it is possible that the relatively small shifts observed are a result of several distinct processes, of which ligand binding may be one.

Due to the low affinity of the interaction between tau K18^M^ and Cl-NQTrp, determination of more detailed structural data remains a challenge. However, the study of tau K18^M^ and Cl-NQTrp interactions indicates that NMR may be one of the least artefact-prone and most robust techniques for characterizing such IDP–ligand interactions, provided the tested compounds exhibit satisfactory solubility profiles. SPR and MST methods also provide orthogonal approaches, allowing the use of limited solubility compounds, although the amount of structural data obtained may be limited. Furthermore, these studies highlight that care must be taken when interpreting biophysical data relating to low affinity and transient interactions, and that while a simple interpretation of a single result is not always possible the careful use of orthogonal experiments and techniques can provide a robust characterization of an IDP–ligand interaction.

## 4. Materials and Methods 

### 4.1. Materials

Initial batch of ^15^N-labeled 6xHis-tau K18(C291S, C322S), or tau K18^M^, and 9 literature compounds were kindly provided by Servier (Paris, France). All reagents and solvents were obtained from commercial vendors unless stated otherwise.

### 4.2. Protein Expression and Purification

Codon-optimized genes were ordered from GenScript (Piscataway, NJ, USA) in pET24a expression vector with NdeI and XhoI restriction sites. Protein concentrations were determined using Pierce BCA assay kit (Thermofisher Scientific, Vilnius, Lithuania). Protein intact mass was determined using HPLC-MS (Agilent, Santa Clara, CA, USA) (Supplementary Data). Protein purification was performed using AKTA FPLC system (GE Healthcare, Uppsala, Sweden).

#### 4.2.1. 6xHis-tau K18(C291S, C322S)

The protein-coding plasmid was transformed to *E. coli* BL21(DE3) cells and cultured at 37 °C in flasks containing Luria–Bertani (LB) with 50 µg/mL kanamycin. The expression was induced at OD_600_ = 0.8 with 1 mM final concentration of IPTG for 4 h at 37 °C. After expression, the cells were pelleted at 5000× *g* for 20 min and stored at −80 °C. For producing protein for NMR studies, M9 media supplemented with [^13^C] D-glucose (2 g/L) and/or ^15^NH_4_Cl (1 g/L) was used.

Cell pellets were resuspended in ice-cold lysis buffer (50 mM NaP_i_ pH 7.5, 500 mM NaCl, 10 mM imidazole), supplemented with cOmplete Protease Inhibitor Cocktail EDTA-free tablets, and lysed using French press homogenization system (Homogenising Systems Limited, Stansted, UK). The lysate was then incubated at 90 °C for 15 min under mild stirring. Following this, the lysate was cooled down in ice bath for 10 min and further clarified by centrifugation at 40,000× *g* for 1 h. The supernatant was loaded onto a 1 mL HisTrap FF column (GE Healthcare) equilibrated with 25 mM NaP_i_ pH 7.5, 500 mM NaCl, 10 mM imidazole and the protein was eluted using a 3-step gradient (5, 20 and 100%) of 25 mM NaP_i_ pH 7.5, 500 mM NaCl, 500 mM imidazole. Fractions containing the protein were desalted to 25 mM NaP_i_ pH 7.0, 25 mM NaCl, 1 mM EDTA using HiPrep Desalt 26/10 column and loaded onto pre-equilibrated with desalting buffer 5 mL HiTrap SP FF column. The protein was eluted with gradient over 10 CV from 0 to 100% of 25 mM NaP_i_ pH 7.0, 1 M NaCl, 1 mM EDTA. Protein-containing fractions were further purified using HiPrep Superdex 16/60 75 PG column in 25 mM NaP_i_ pH 6.6, 150 mM NaCl, 0.1 mM EDTA, snapfrozen in liquid N_2_ and stored at −80 °C.

#### 4.2.2. 6xHis-TEV-AviTag-tau K18(C291S, C322S)

The protein was expressed as described for non-labeled tau K18^M^ construct. After this, the protein was purified using the same protocol as for non-labeled protein until after the elution from HisTrap FF column. Protein-containing fractions were then desalted to 25 mM NaP_i_ pH 7.5, 500 mM NaCl, 10 mM imidazole using HiPrep 26/10 column. The sample was supplemented with 2.5 mg of TEV protease (in-house) and incubated at 4 °C overnight. After incubation, the sample was further purified by loading the mixture on 1 mL HisTrap FF column in 25 mM NaP_i_ pH 7.5, 500 mM NaCl, 10 mM. After eluting the protein with 3 step-gradient (5, 20, 100%) of 25 mM NaP_i_ pH 7.5, 500 mM NaCl, 500 mM imidazole, cleaved protein-containing fractions were further purified using HiPrep Superdex 16/60 75 PG column in 25 mM NaP_i_ pH 6.6, 150 mM NaCl. The pure protein samples were then pooled, desalted to 50 mM bicine pH 8.3 and had its concentration adjusted to 40 µM. This was followed by the addition of final concentration of 10 mM ATP, 100 µM D-biotin, 10 mM MgCl_2_, and 5 µg of BirA (in-house) (per 10 nmol of AviTag-tau K18(C291S, C322S). The mixture was incubated at room temperature for 2 h and biotinylation efficiency was determined by HPLC-MS. After biotinylation, the sample was further purified using HiPrep Superdex 16/60 75 PG, pre-equilibrated with 25 mM NaP_i_ pH 6.6, 150 mM NaCl, snapfrozen in liquid N_2_ and stored at −80 °C.

### 4.3. Compound Synthesis

(2S)-2-[(3-chloro-1,4-dioxonaphthalen-2-yl)amino]-3-(1H-indol-3-yl)propanoic acid (Cl-NQTrp) and its near-neighbors (NNs) were synthesized using adapted one-step synthesis protocol [52]. Compound purity and structural integrity was assessed using ^1^H NMR and HPLC-MS (Supplementary Data).

### 4.4. Nuclear Magnetic Resonance

Protein and ligand NMR data were obtained using Bruker AVANCE 600 MHz spectrometer(Bruker BioSpin, Fällanden, Switzerland) equipped with a BACS-120 sample changer and QCI-F cryoprobe in 50 mM NaP_i_ pH 6.6, 25 mM NaCl, 100 µM DSS, 5% D_2_O, 4% d_6_-DMSO at 298 K, unless stated otherwise. Bruker DPX-400 spectrometer (Bruker BioSpin, Fällanden, Switzerland) was used to assess structural integrity of organic compounds in d_6_-DMSO at 298 K. The NMR data were processed and analyzed using TopSpin 4.0.2 (Bruker BioSpin, Fällanden, Switzerland), MNova 11.0 (MestreLab Research, Santiago de Compostela, Spain) and Dynamics Center 2.5.4 (Bruker BioSpin, Fällanden, Switzerland). Experimental NMR parameters can be found in Table S1.

### 4.5. Surface Plasmon Resonance

Immobilization of target protein was performed in 50 mM HEPES pH 7.4, 150 mM NaCl, 0.05% Tween-20 at 20 °C using Biacore T200 (GE Healthcare, Uppsala, Sweden). For biotin-SA coupling, the surface of S Series Sensor Chip SA (GE Healthcare, Uppsala, Sweden) was first conditioned with three consecutive 1 min injections of 1 M NaCl + 50 mM NaOH at 10 µL/min. This was followed by injection of 100 nM of biotinylated protein at 5 µL/min until immobilization level of 1000 RU. Eight startup cycles were used for stabilizing the surface after the immobilization.

Single-cycle kinetics experiments were performed as 1:1 serial dilution concentrations of the ligands with 45 s association and 60 s dissociation times at 30 µL/min at 20 °C in 50 mM HEPES pH 7.4, 150 mM NaCl, 0.05% Tween-20, 4% DMSO. The ligand concentrations used were based on solubility studies by NMR and literature data. Blank injections were also used for double referencing. Solvent correction was performed with 8 point samples at from 3.5 to 4.8% DMSO. The flow system was washed with 50% DMSO after each cycle.

Theoretical *R_max_* was calculated using the following formula:

$$R_{max}=\frac{Immobilization\;level\;of\;target\;protein\;\left(RU\right)}{MW\;of\;immobilized\;protein\;\left(Da\right)}*MW\;ofligand\;\left(Da\right)*1\left(valency\right)$$

Experimental data was analyzed using Biacore Insight Evaluation Software (GE Healthcare, Uppsala, Sweden) and SimFit (https://www.simfit.org.uk/).

### 4.6. Microscale Thermophoresis 

The experiments were performed on Monolith NT Automated (NanoTemper, Munich, Germany) machine with the following parameters:

- MST power = 40%
- Excitation power = 15%
- Temperature = 20 °C
- Acquisition mode = Pico-Red

The data was processed using MO.Affinity analysis v2.2.4 (NanoTemper). The change in fluorescence was used to fit the data to either 1:1 K_D_ model or Hills equation.

For protein–ligand interaction measurements using dye labeling, 6xHis-tagged protein was labeled with RED-Tris dye following manufacturer’s protocol. Following this, an appropriate number of the labeled protein samples with the ligand were prepared by mixing 1 µL of ligand in 100% DMSO with 24 µL of labeled protein in 50 mM HEPES pH 7.4, 150 mM NaCl, 0.05% Tween 20. Final DMSO concentration = 4%. The samples were incubated for 30 min, loaded into premium-coated capillaries and measured. The ligand concentrations used were based on solubility studies by NMR and literature data

For non-labeled approach, 24 samples of tau K18^M^ were prepared from 5.9 to 1000 nM as 1.25× dilutions in the presence of 100 nM of compound **1** (Methylene Blue) in 50 mM HEPES pH 7.4, 150 mM NaCl, 0.05% Tween 20, 4% DMSO.

### 4.7. Molecular Dynamics

The simulation system of Cl-NQTrp was prepared as follows. To prepare Cl-NQTrp, the compound titratable groups were assigned standard protonation states at pH = 7 and the 2D to 3D conversion was done by MacroModel [53]. After that, the simulation system of Cl-NQTrp was built with the Maestro graphical user interface designed for Desmond [54], distributed by Schrödinger tools (Schrödinger, New York, NY, USA). The default setup parameters were kept which used the NPT ensemble with explicit SPC waters and added counterions to neutralize the overall charge of the system. 

The MD was performed by Desmond at constant temperature (300 K) and pressure (1.01 bar), and coordinates saved every 4.8 picoseconds, keeping the default settings. The OPLS3 force-field was used [55]. Cl-NQTrp was simulated for 1.0 μs free in aqueous solution. To analyze the simulation results, the snapshots were extracted from Cl-NQTrp MD with the Schrödinger tools.

## 5. Conclusions

We have characterized the interaction of a literature ligand, Cl-NQTrp, with the model IDP tau K18^M^, using a range of biophysical techniques including protein- and ligand-observed NMR, SPR, and MST. Close near neighbors of the Cl-NQTrp parent compound were synthesized and observed to bind to the same site in tau K18^M^ with a range of affinities. The studies identified issues with the applicability and artefacts with all biophysical methods used. While NMR proved the most detailed and robust data about IDP–ligand interactions, complementary biophysical methods, such as SPR and MST, should be used, particularly if compound solubility is limited. Additional studies would be necessary for a more complete structural characterization of the interactions in the tau K18^M^–Cl-NQTrp.

## Supplementary Materials

The following are available online at https://www.mdpi.com/1422-0067/21/15/5257/s1, Figure S1: 2D [^1^H-^15^N] SF-HMQC spectra of ^15^N-labelled tau K18^M^ in the presence and absence of literature compounds, Figure S2: 2D [1H-13C] HSQC spectra of 13C,15N-labelled tau K18M in the presence and absence of compound 6, Figure S3: Kinetic SPR binding curves and steady-state affinity graphs for literature compounds, Figure S3: Kinetic SPR binding curves and steady-state affinity graphs for literature compounds, Figure S4: MST dose-response data for literature compounds to dye-labelled tau K18M, Figure S5: Investigation of potential Cl-NQTrp covalent adducts to tau K18M using MS and NMR, Figure S6: 2D [1H-15N] SF-HMQC spectra of 15N tau K18M in the presence of 2 mM Cl-NQTrp at different concentrations of NaCl., Figure S7. The observed CSPs for 15N tau K18M in the presence of ethanolamine, acetic.

## Abbreviations

CPMG	Carr–Purcell–Meiboom–Gill
CSP	Chemical shift perturbation
DOSY	Diffusion-ordered spectroscopy
IDP	Intrinsically disordered protein
MST	Microscale thermophoresis
PHF	Paired helical filament
SPR	Surface plasmon resonance
STD	Saturation transfer difference
Water-LOGSY	Water–ligand observed via gradient spectroscopy
