# Supplementary material for: Exploring IDP–Ligand Interactions: Tau K18 as a Test Case
Source: Int J Mol Sci. 2020 Jul 24;21(15):5257. doi: 10.3390/ijms21155257 (PMC7432903; doi:10.3390/ijms21155257)
Supplement: Supplementary file 1 [file ijms-21-05257-s001.zip › Supplementary Data_revised.pdf]

Supplementary Data

Details of protein constructs

6xHis-tau K18(C291S,C322S)

MGHHHHHHQTAPVPMPDLKNVKSKIGSTENLKHQPGGGKVQIINKKLDLSNVQSKSGSKDNI
KHVPGGGSVQIVYKPVDLSKVTSKSGSLGNIHHKPGGGQVEVKSEKLDFKDRVQSKIGSLDN
ITHVPGGGNKKIE

6xHis-TEV-AviTag-tau K18(C291S,C322S)

MHHHHHHENLYFQGS**GLNDIFEAQKIEWHE**GSQTAPVPMPDLKNVKSKIGSTENLKHQPGGG
KVQIINKKLDLSNVQSKSGSKDNIKHVPGGGSVQIVYKPVDLSKVTSKSGSLGNIHHKPGGG
QVEVKSEKLDFKDRVQSKIGSLDNITHVPGGGNKKIE

Affinity tag is marked in cyan, whereas the mutations from Cys to Ser are marked in yellow. Enzymatic cleavage sequence is underlined. AviTag is **bolded**.

Supplementary figures and tables

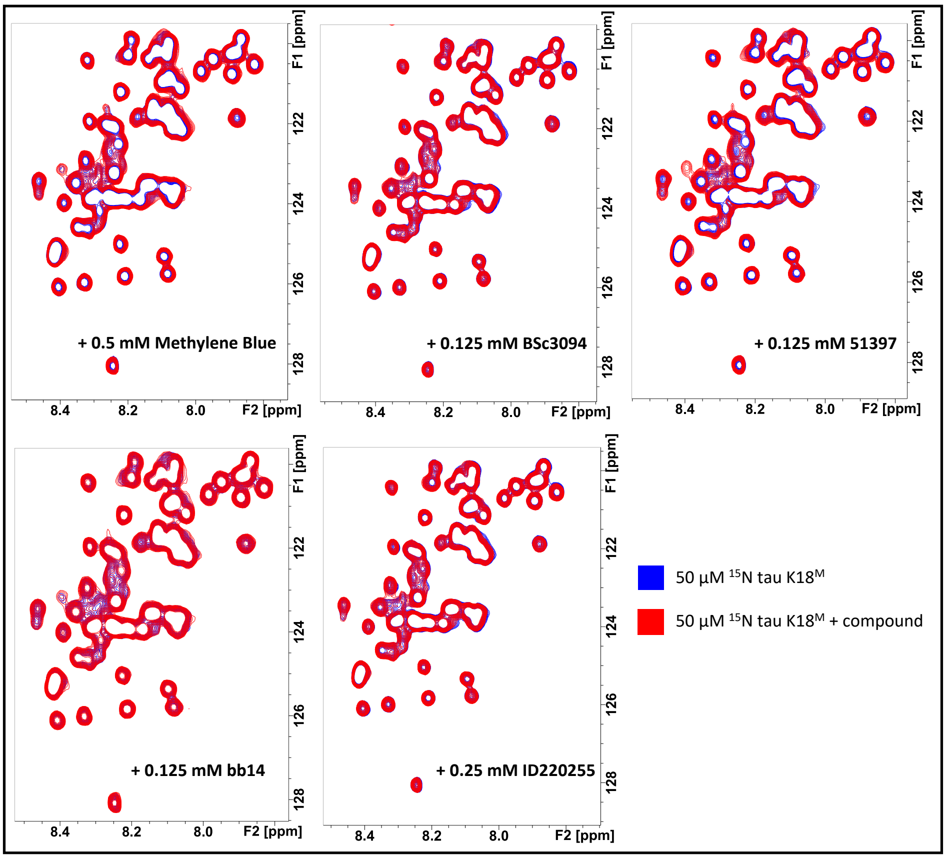

Figure S1. 2D [^{1}H-^{15}N] SF-HMQC spectra of ^{15}N-labelled tau K18^{M} in the presence and absence of literature compounds.

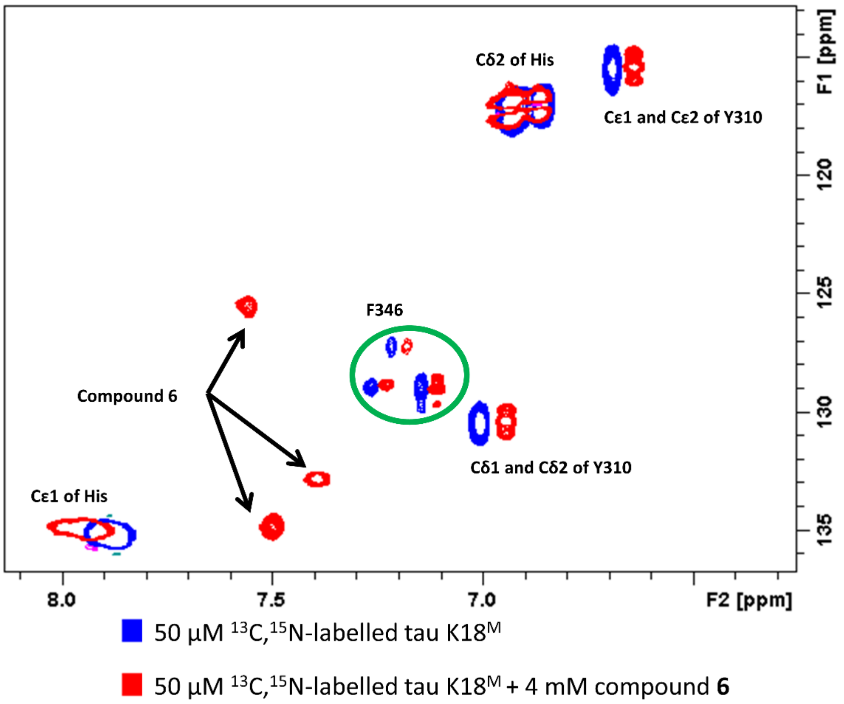

Figure S2. 2D [^{1}H-^{13}C] HSQC spectra of ^{13}C,^{15}N-labelled tau K18^M in the presence and absence of compound **6**.

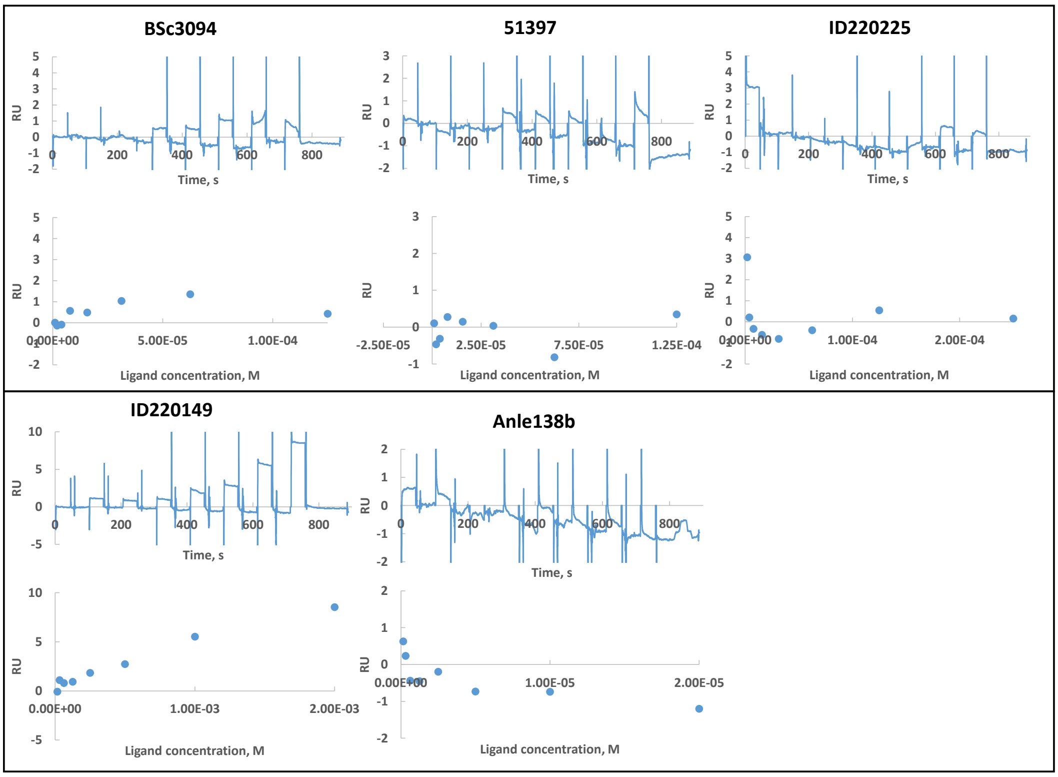

Figure S3. Kinetic SPR binding curves and steady-state affinity graphs for literature compounds. Immobilization level: 1000 RU of biotinylated AviTag-tau K18^M.

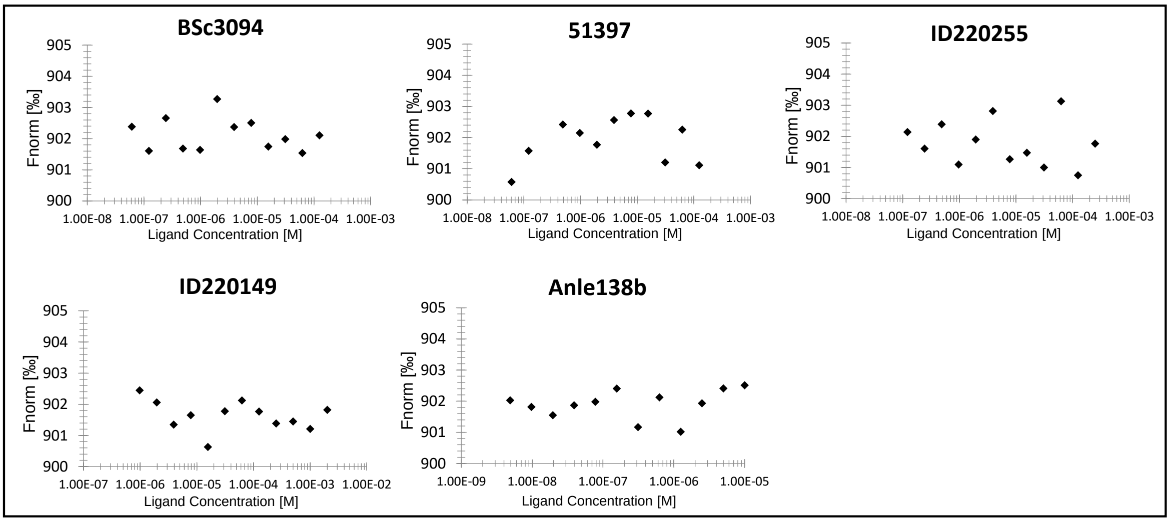

Figure S4. MST dose-response data for literature compounds to dye-labelled tau K18^{M}.

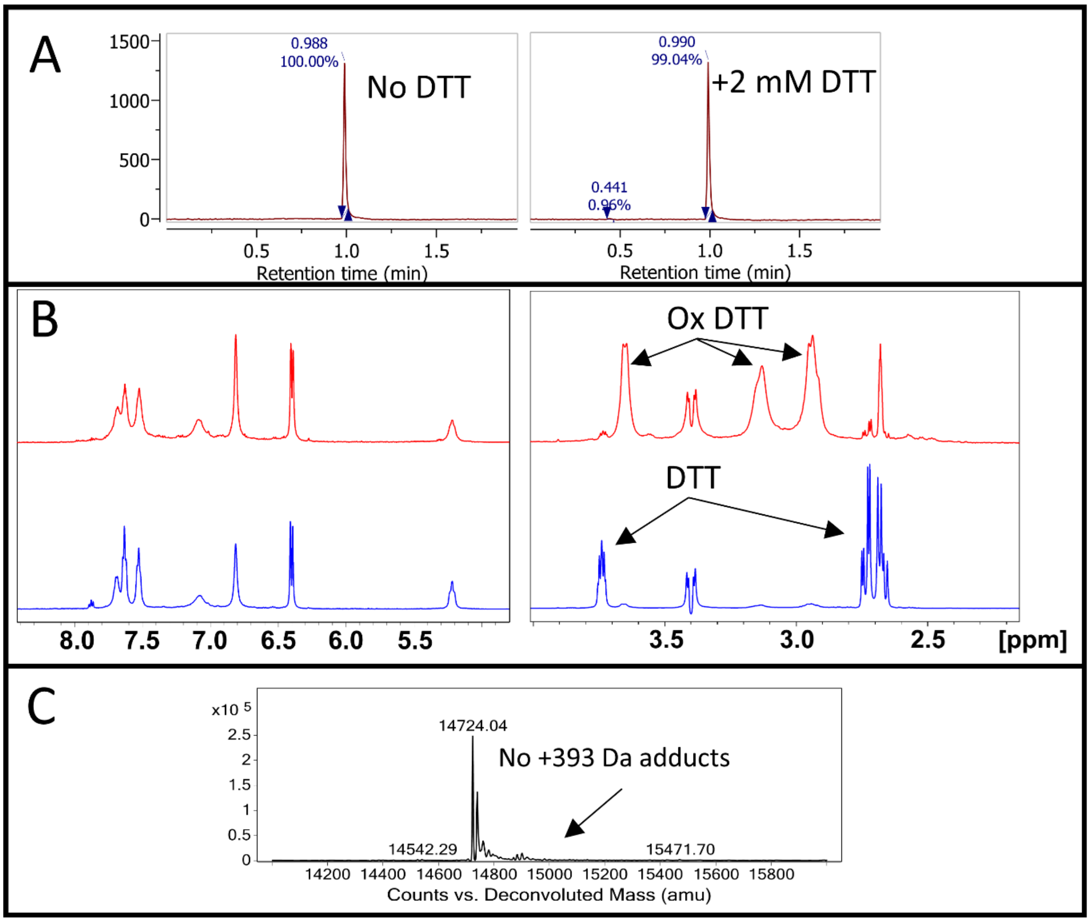

Figure S5. Investigation of potential Cl-NQTrp covalent adducts to tau K18^{M} using MS and NMR. **A.** HPLC-MS data indicating no elution pattern changes after 1 mM Cl-NQTrp was incubated for 72 hours in the presence of 2 mM dithiothreitol (DTT) in 50 mM NaP$_i$ pH 6.6, 25 mM NaCl. **B.** NMR data showing no indication of structural changes for Cl-NQTrp after 72 h incubation with 2 mM DTT in 50 mM NaP$_i$ pH 6.6, 25 mM NaCl. **C.** HPLC-MS data showing no indication of any covalent adducts to 50 µM ^{15}N labelled tau K18^{M} after 2 hour incubation with 1 mM Cl-NQTrp in 50 mM NaP$_i$ pH 6.6, 25 mM NaCl.

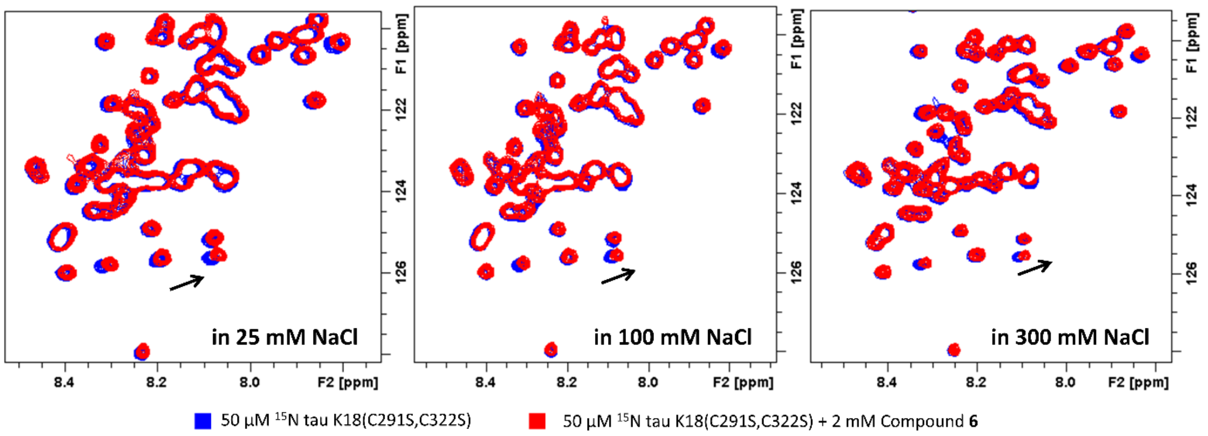

Figure S6. 2D [^{1}H-^{15}N] SF-HMQC spectra of ^{15}N tau K18^{M} in the presence of 2 mM Cl-NQTrp at different concentrations of NaCl. The black arrows denote the vector of observed CSPs.

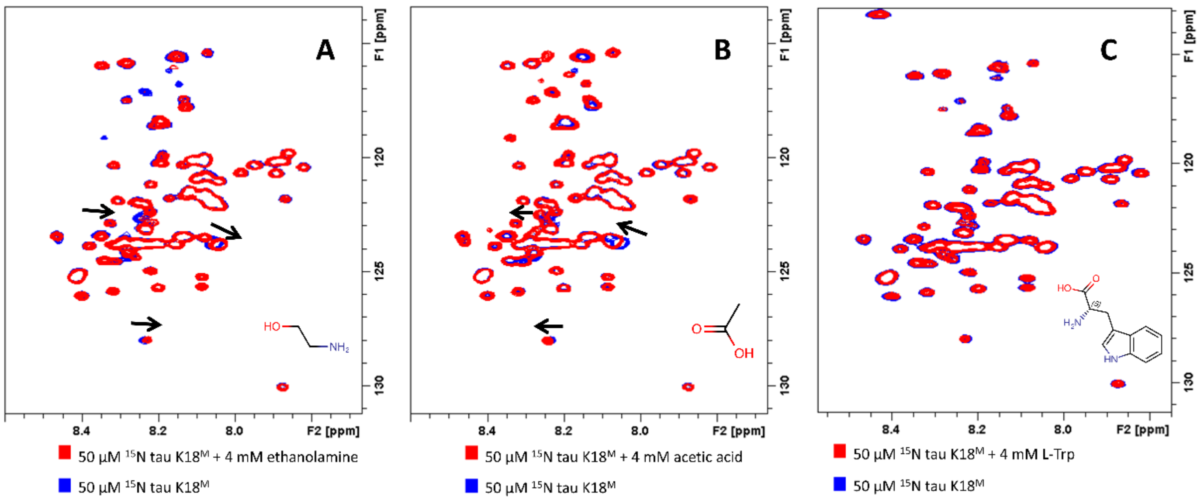

Figure S7. The observed CSPs for ^{15}N tau K18^{M} in the presence of ethanolamine (**A**), acetic acid (**B**) and L-Trp (**C**) in 2D [^{1}H-^{15}N] SF-HMQC spectra. The black arrows denote the vector of observed CSPs.

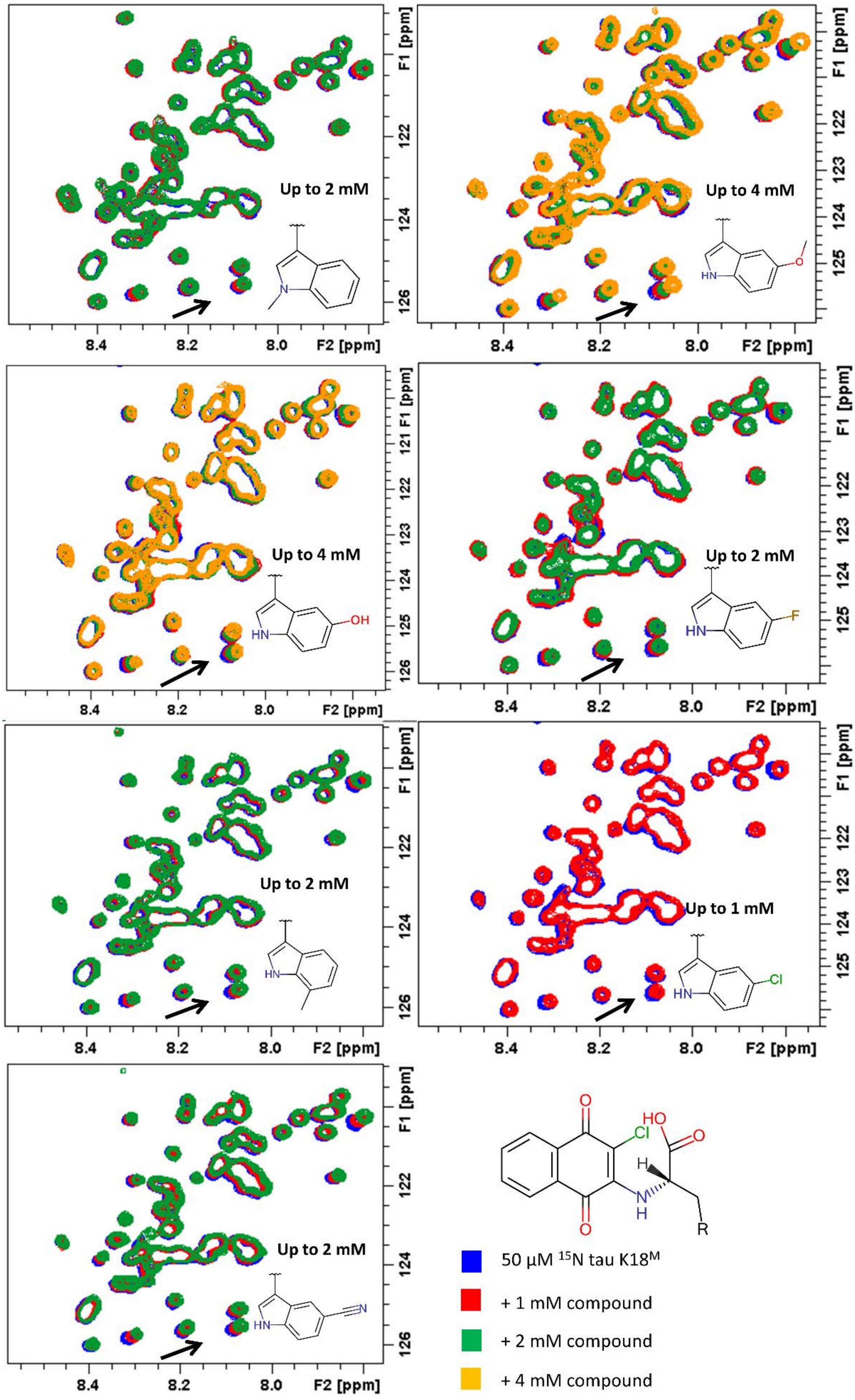

Figure S8. 2D [^{1}H-^{15}N] SF-HMQC spectra in the presence of different concentrations of NNs with ^{15}N-labelled 50 μM tau K18^{M}. The black arrows denote the vector of observed CSPs.

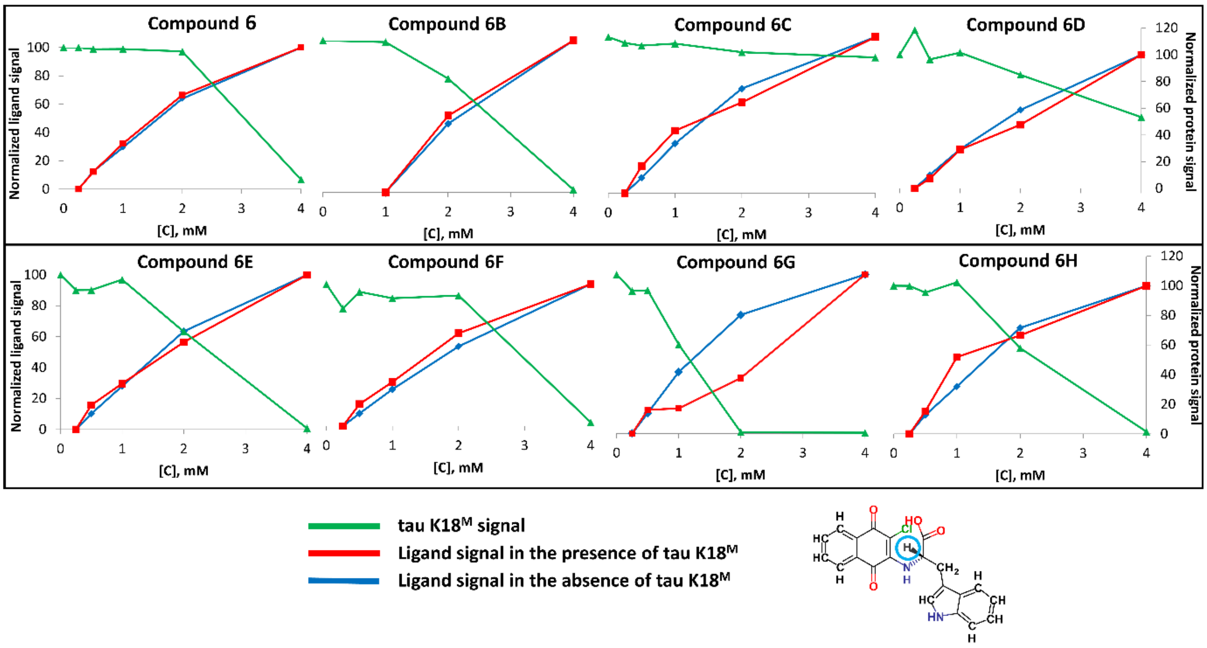

Figure S9. A comparison of tau K18^{M} solubility profile in the presence of Cl-NQTrp and its NNs. The well-resolved C-H proton (circled in blue) and protein methyl envelope (0.76-1 ppm) were used to track ligand and tau K18^{M} signals, respectively.

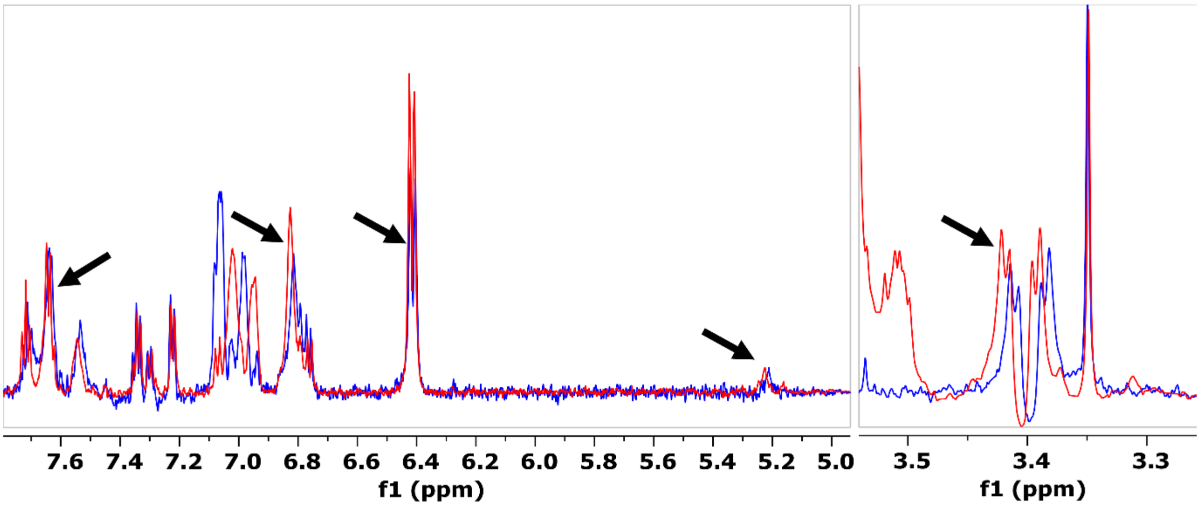

Figure S10. 1D ^{1}H CPMG-filtered NMR spectra of 1 mM compound **6D** with 50 µM tau K18^{M} in the absence (blue) and presence (red) of 0.2% Tween-20 (τ = 200 ms) indicating the lack of significant signal recovery in the presence of detergent. The black arrows denote ligand-specific signals.

Table S1. The experimental parameters used for NMR experiments.

Experiment	NS	SW, ppm	FID	Extra parameters	PULPROG
1D ^{1}H	64	16	32768	-	zgesgp
1D ^{1}H CPMG-filtered	256	16	32768	τ = variable	bdcpmgesdf.rh
1D ^{1}H STD	128	16	32768	On-resonance = 0.7 ppm, off-resonance = -27.6 ppm	bdSTDesgpdf.rh
1D ^{1}H water-LOGSY	32	16	32768	-	bdLOGSYesgpdf.rh
1D ^{19}F NMR	256	237.2	262144	-	zgig30
Pseudo 2D DOSY	32	F1 = 10, F2 = 20	F1 = 16, F2 = 2048	Δ = 50 ms δ = 8 ms	bd-ledbpgppr2s
2D [^{1}H-^{15}N] SF-HMQC	32	F1 = 31.8, F2 = 16	F1 = 128, F2 = 2048	NUS = 50%	sfhmqcf3gpph
2D [^{1}H-^{13}C] HSQC	16	F1 = 31.8, F2 = 16	F1 = 128, F2 = 2048	NUS = 50%	Chsqc.rh

Table S2. T_2 relaxation values for tryptophan methylene group at different concentrations of compound **6** and its NNs in the absence and presence of tau K18^M.

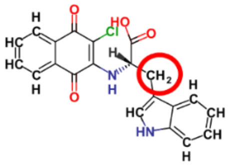

Compound	T_2 relaxation time for methylene group, ms			
	Without protein		With protein	
	100 µM	1000 µM	100 µM	1000 µM
6(Cl-NQTrp)	50 +/- 10	39 +/- 12	9 +/- 2	18 +/- 6
6B	41 +/- 6	33 +/- 8	13 +/- 4	14 +/- 2
6D	36 +/- 6	26 +/- 5	4 +/- 1	13 +/- 2
6E	24 +/- 3	22 +/- 3	8 +/- 2	12 +/- 2
6F	27 +/- 5	15 +/- 1	10 +/- 2	8 +/- 2
6G	34 +/- 6	34 +/- 5	13 +/- 2	18 +/- 5
6H	53 +/- 11	36 +/- 7	11 +/- 6	26 +/- 4
L-Trp	557+/- 84	559 +/- 63	-	-

HPLC-MS and HRMS-ESI

Exact protein molecular mass was determined using Agilent HPLC 1290 series coupled to a 6230 TOF (positive ESI mode) with Agilent MassHunter software. The column (BioResolve RP mAB polyphenyl, 150 x 2.1 mm, 2.7 µm, Waters) was run under gradient condition at pH = 4 (5 to 95% A to B, A = 0.08% formic acid 0.01% TFA, B = MeCN+ 0.08% formic acid, 0.01% TFA) over 5 min at a rate of 0.8 mL/min at 55 ^{o}C).

LMW organic compound studies were performed on Agilent 1200 SL series instrument linked to an Agilent MSD 6140 single quadrupole with an ESI-APCI multimode source or Agilent 1290 Infinity II series instrument connected to an Agilent TOF 6230 with an ESI-jet stream source. Column: Thermo Accucore 2.6 µm, C18, 50 mm x 2.1 mm at 55 ^{o}C. Injection volume: 1 µL. Elution: 95% A / 5% B to 5% A / 95% B using HPLC-grade solvents.

- Solvent A: 10 mM aqueous ammonium formate + 0.04% (v/v) formic acid
- Solvent B: Acetonitrile + 5.3% (v/v) Solvent A + 0.04% (v/v) formic acid.

**Time (min)**	**LC-MS-V-B1**		**Flow (mL/min)**
	**Solvent A (%)**	**Solvent B (%)**	
0	95	5	1.1
0.12	95	5	1.3
1.30	5	95	1.3
1.35	5	95	1.6
1.85	5	95	1.6
1.90	5	95	1.3
1.95	95	5	1.3

Compound synthesis

All solvents and reagents were used as obtained from commercial vendors. Purification steps were carried out using HPLC-grade solvents.

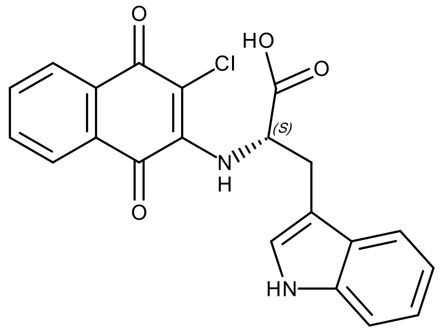

Compound 6

To a stirred solution of 2,3-dichloro-1,4-naphthoquinone (113.52 mg, 0.5 mmol) in 19 mL absolute EtOH was added dropwise (2S)-2-amino-3-(1H-indol-3-yl)propanoic acid (62.5 mg, 0.3 mmol) which was previously dissolved in 1 mL of 1 M KOH in distilled H_2O. The mixture was stirred at 30 ^oC overnight with a mounted condenser. After incubation, the sample was acidified dropwise by addition of 2 M HCl until pH = 2, as determined with pH paper indicator. Following this, the compound was purified by automated flash chromatography (Combiflash Rf, Silica 40g RediSep column) eluting with 0 to 100% ethyl acetate in heptane. Pure fractions, as determined by HPLC-MS, were further purified by reverse phase automated flash chromatography (Combiflash Rf, C18 50g Gold RediSep column) eluting with 10 to 100% acetonitrile in pH 4 water. Freeze-drying yielded red powder of **(2S)-2-[(3-chloro-1,4-dioxonaphthalen-2-yl)amino]-3-(1H-indol-3-yl) propanoic acid.**

Yield: 67.35 mg or 55.74%. **NMR:** ^1H NMR (399 MHz, DMSO-d_6) δ 13.35 (s, 1H), 10.96 (d, J = 2.4 Hz, 1H), 8.00 – 7.93 (m, 1H), 7.89 (d, J = 7.7 Hz, 1H), 7.83 (td, J = 7.5, 1.4 Hz, 1H), 7.75 (td, J = 7.5, 1.4 Hz, 1H), 7.51 (d, J = 7.9 Hz, 1H), 7.30 (d, J = 8.1 Hz, 1H), 7.22 (d, J = 2.4 Hz, 1H), 7.04 (ddd, J = 8.2, 7.0, 1.2 Hz, 1H), 6.98 – 6.89 (m, 1H), 6.58 (d, J = 24.1 Hz, 1H), 5.33 (d, J = 7.2 Hz, 1H), 3.40 (t, J = 5.2 Hz, 2H). **HPLC-MS**: ($C_{21}H_{15}ClN_2O_4$) 394.98 $[M+H]^+$; RT 1.083. **HRMS-ESI:** calculated for $C_{21}H_{15}ClN_2O_4$: 394.072, found 394.0745

The following derivatives were prepared using analogous protocol.

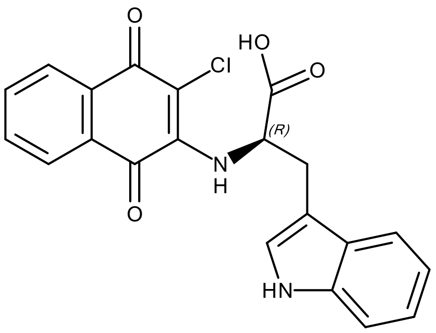

***R*-enantiomer of Compound 6 (6A)**

(2R)-2-[(3-chloro-1,4-dioxonaphthalen-2-yl)amino]-3-(1H-indol-3-yl)propanoic acid. Obtained from 2,3-dichloro-1,4-naphthoquinone and (2R)-2-amino-3-(1H-indol-3-yl)propanoic acid. **Yield:** 55 mg or 46.51%. **NMR:** ^{1}H NMR (399 MHz, DMSO-d6) δ 13.33 (s, 1H), 10.97 (d, J = 2.5 Hz, 1H), 7.96 (d, J = 7.6 Hz, 1H), 7.89 (d, J = 7.5 Hz, 1H), 7.83 (td, J = 7.5, 1.4 Hz, 1H), 7.74 (td, J = 7.5, 1.4 Hz, 1H), 7.51 (d, J = 7.9 Hz, 1H), 7.33 – 7.26 (m, 1H), 7.22 (d, J = 2.4 Hz, 1H), 7.03 (ddd, J = 8.1, 7.0, 1.2 Hz, 1H), 6.98 – 6.89 (m, 1H), 6.56 (s, 1H), 5.33 (d, J = 7.3 Hz, 1H), 3.40 (t, J = 5.6 Hz, 2H). **HPLC-MS:** ($C_{21}H_{15}ClN_2O_4$) 394.98 $[M+H]^+$; RT 1.105. **HRMS-ESI:** calculated for $C_{21}H_{15}ClN_2O_4$: 394.077, found 394.0752.

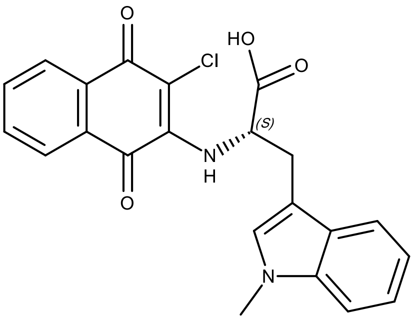

Compound 6B

(2S)-2-[(3-chloro-1,4-dioxonaphthalen-2-yl)amino]-3-(1-methylindol-3-yl)propanoic acid. Obtained from 2,3-dichloro-1,4-naphthoquinone and (2S)-2-amino-3-(1-methylindol-3-yl)propanoic acid. **Yield**: 45 mg or 36.63%. **NMR:** ^{1}H NMR (399 MHz, DMSO-d6) δ 13.31 (s, 1H), 7.87 (d, J = 7.6 Hz, 1H), 7.75 (td, J = 7.4, 1.4 Hz, 1H), 7.72 (s, 1H), 7.66 (td, J = 7.5, 1.3 Hz, 1H), 7.43 (d, J = 7.9 Hz, 1H), 7.22 (d, J = 8.2 Hz, 1H), 7.10 (s, 1H), 7.05 – 6.97 (m, 1H), 6.94 – 6.85 (m, 1H), 6.50 (s, 1H), 5.26 (dt, J = 8.4, 5.8 Hz, 1H), 3.57 (s, 3H), 3.30 (d, J = 5.7 Hz, 2H). **HPLC-MS**: ($C_{22}H_{17}ClN_2O_4$) 408.84 $[M+H]^+$ RT 1.22. **HRMS-ESI**: calculated for $C_{22}H_{17}ClN_2O_4$: 408.0877, found: 408.0894.

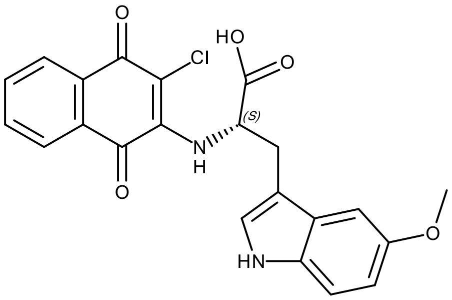

Compound 6C

(2S)-2-[(3-chloro-1,4-dioxonaphthalen-2-yl)amino]-3-(5-methoxy-1H-indol-3-yl) propanoic acid. Obtained from 2,3-dichloro-1,4-naphthoquinone and (2S)-2-amino-3- (5-methoxy-1H-indol-3-yl)propanoic acid**. Yield:** 73 mg or 57.14%. **NMR:** ^{1}H NMR (399 MHz, DMSO-d_6) δ 13.35 (s, 1H), 10.80 (d, J = 2.6 Hz, 1H), 7.96 (d, J = 7.6 Hz, 1H), 7.88 (d, J = 7.6 Hz, 1H), 7.83 (td, J = 7.6, 1.4 Hz, 1H), 7.74 (td, J = 7.5, 1.4 Hz, 1H), 7.24 – 7.07 (m, 2H), 6.98 (d, J = 2.4 Hz, 1H), 6.65 (dd, J = 8.7, 2.4 Hz, 2H), 5.60 – 5.10 (m, 1H), 3.69 (s, 3H), 3.37 (d, J = 6.8 Hz, 2H). **HPLC-MS:** ($C_{22}H_{17}ClN_2O_5$) 424.99 $[M+H]^+$; RT 1.08. **HRMS-ESI:** calculated for $C_{22}H_{17}ClN_2O_5$: 424.0826, found 424.0855.

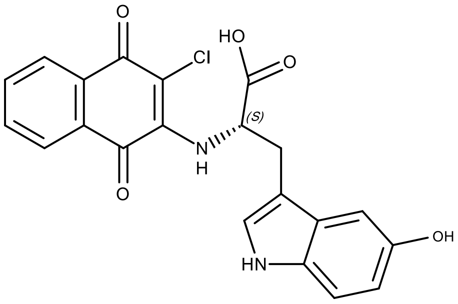

Compound 6D

(2S)-2-[(3-chloro-1,4-dioxonaphthalen-2-yl)amino]-3-(5-hydroxy-1H-indol-3-yl) propanoic acid. Obtained from 2,3-dichloro-1,4-naphthoquinone and (2S)-2-amino-3- (5-hydroxy-1H-indol-3-yl)propanoic acid. **Yield**: 49.7 mg or 40.34%. **NMR:** ^{1}H NMR (399 MHz, DMSO-d_6) δ 13.30 (s, 1H), 10.65 (d, J = 2.5 Hz, 1H), 8.62 (s, 1H), 7.96 (d, J = 7.6 Hz, 1H), 7.89 (s, 1H), 7.83 (td, J = 7.5, 1.4 Hz, 1H), 7.74 (td, J = 7.5, 1.4 Hz, 1H), 7.14 – 7.05 (m, 2H), 6.83 (d, J = 2.3 Hz, 1H), 6.57 (dd, J = 8.6, 2.3 Hz, 2H), 5.31 – 5.23 (m, 1H), 3.30 (d, J = 5.8 Hz, 2H). **HPLC-MS:** ($C_{21}H_{15}ClN_2O_5$) 410.97 $[M+H]^+$; RT 0.93. **HRMS-ESI:** calculated for $C_{21}H_{15}ClN_2O_5$: 410.0669, found 410.0736.

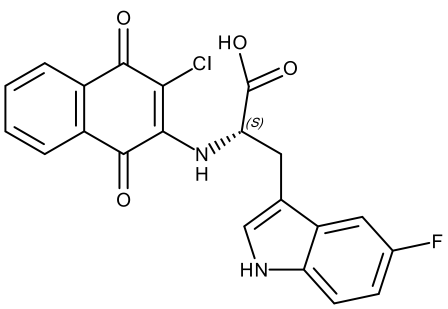

Compound 6E

(2S)-2-[(3-chloro-1,4-dioxonaphthalen-2-yl)amino]-3-(5-fluoro-1H-indol-3-yl)propanoic acid. Obtained from 2,3-dichloro-1,4-naphthoquinone and (2S)-2-amino-3- (5-fluoro-1H-indol-3-yl)propanoic acid. **Yield:** 41.55 mg or 33.54%. **NMR:** ^{1}H NMR (399 MHz, DMSO-d_6) δ 13.28 (s, 1H), 11.06 (d, J = 2.5 Hz, 1H), 7.96 (dd, J = 7.7, 1.3 Hz, 1H), 7.89 (d, J = 7.7 Hz, 1H), 7.83 (td, J = 7.5, 1.4 Hz, 1H), 7.75 (td, J = 7.5, 1.4 Hz, 1H), 7.29 (d, J = 2.7 Hz, 1H), 7.28 – 7.25 (m, 1H), 7.23 (dd, J = 10.1, 2.5 Hz, 1H), 6.86 (td, J = 9.2, 2.5 Hz, 1H), 6.60 (d, J = 36.0 Hz, 1H), 5.33 (q, J = 6.3 Hz, 1H), 3.41 (d, J = 14.9 Hz, 2H). **HPLC-MS:** ($C_{21}H_{14}ClFN_2O_4$) 412.99 $[M+H]^+$; RT 1.11. **HRMS-ESI:** calculated for $C_{21}H_{14}ClFN_2O_4$: 412.0626, found 412.0698.

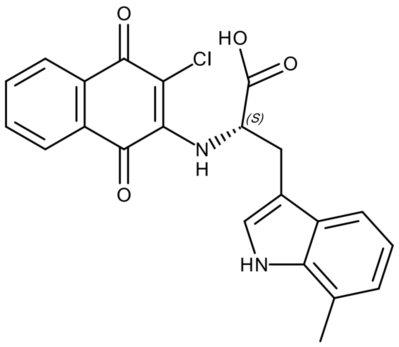

Compound 6F

(2S)-2-[(3-chloro-1,4-dioxonaphthalen-2-yl)amino]-3-(7-methyl-1H-indol-3-yl)propanoic acid. Obtained from 2,3-dichloro-1,4-naphthoquinone and (2S)-2-amino-3- (7-methyl-1H-indol-3-yl)propanoic acid. **Yield:** 59 mg or 48.07%. **NMR:** ^{1}H NMR (399 MHz, DMSO-d_6) δ 13.37 – 13.31 (m, 1H), 10.92 (d, J = 2.5 Hz, 1H), 7.95 (d, J = 7.7 Hz, 1H), 7.90 – 7.78 (m, 2H), 7.73 (td, J = 7.6, 1.3 Hz, 1H), 7.32 (dd, J = 7.3, 1.7 Hz, 1H), 7.19 (d, J = 2.5 Hz, 1H), 6.89 – 6.78 (m, 2H), 6.61 (s, 1H), 5.32 (d, J = 7.2 Hz, 1H), 3.38 (d, J = 5.7 Hz, 2H), 2.37 (s, 3H). **HPLC-MS:** ($C_{22}H_{17}ClN_2O_4$) 408.99 $[M+H]^+$; RT 1.16. **HRMS-ESI:** calculated for $C_{22}H_{17}ClN_2O_4$: 408.0877, found 408.0907.

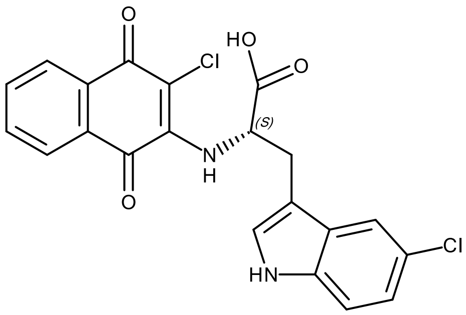

Compound 6G

(2S)-2-[(3-chloro-1,4-dioxonaphthalen-2-yl)amino]-3-(5-chloro-1H-indol-3-yl)propanoic acid. Obtained from 2,3-dichloro-1,4-naphthoquinone and (2S)-2-amino-3- (5-chloro-1H-indol-3-yl)propanoic acid. **Yield:** 63.5 mg or 49.37%. **NMR:** ^{1}H NMR (399 MHz, DMSO-d_6) δ 13.39 (s, 1H), 11.16 (d, J = 2.5 Hz, 1H), 7.96 (dd, J = 7.6, 1.3 Hz, 1H), 7.89 (d, J = 7.6 Hz, 1H), 7.83 (td, J = 7.5, 1.4 Hz, 1H), 7.75 (td, J = 7.5, 1.3 Hz, 1H), 7.51 (d, J = 2.1 Hz, 1H), 7.29 (d, J = 1.3 Hz, 1H), 7.28 (d, J = 4.9 Hz, 1H), 7.00 (dd, J = 8.6, 2.0 Hz, 1H), 6.61 (d, J = 45.3 Hz, 1H), 5.34 (d, J = 7.1 Hz, 1H), 3.45 – 3.35 (m, 2H). **HPLC-MS:** ($C_{21}H_{14}Cl_2N_2O_4$) 428.97 $[M+H]^+$; RT 1.16. **HRMS-ESI:** calculated for $C_{21}H_{14}Cl_2N_2O_4$: 428.0331, found 428.0403

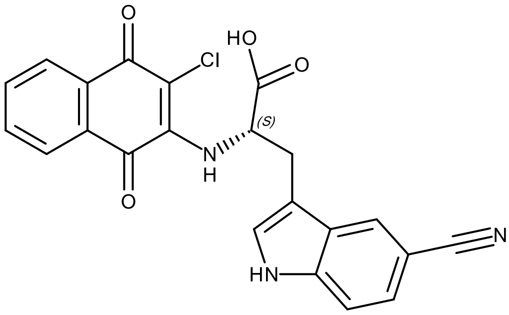

Compound 6H

(2S)-2-[(3-chloro-1,4-dioxonaphthalen-2-yl)amino]-3-(5-cyano-1H-indol-3-yl)propanoic acid. Obtained from 2,3-dichloro-1,4-naphthoquinone and (2S)-2-amino-3- (5-cyano-1H-indol-3-yl)propanoic acid**. Yield:** 69 mg or 54.94%. **NMR:** ^{1}H NMR (399 MHz, DMSO-d_6) δ 13.41 (s, 1H), 11.53 (d, J = 2.4 Hz, 1H), 8.03 (d, J = 1.5 Hz, 1H), 7.96 (dd, J = 7.7, 1.3 Hz, 1H), 7.89 (d, J = 7.6 Hz, 1H), 7.83 (td, J = 7.5, 1.4 Hz, 1H), 7.75 (td, J = 7.5, 1.4 Hz, 1H), 7.47 – 7.41 (m, 2H), 7.35 (dd, J = 8.5, 1.5 Hz, 1H), 6.70 (s, 1H), 5.36 (q, J = 6.6 Hz, 1H), 3.53 – 3.38 (m, 2H). **HPLC-MS:** ($C_{22}H_{14}ClN_3O_4$) 419.99 $[M+H]^+$; RT 1.03. **HRMS-ESI:** calculated for $C_{22}H_{14}ClN_3O_4$: 419.0673, found 419.0701.
