# Supplementary figures and images for: Exploring IDP–Ligand Interactions: Tau K18 as a Test Case
Source: Int J Mol Sci. 2020 Jul 24;21(15):5257. doi: 10.3390/ijms21155257 (PMC7432903; doi:10.3390/ijms21155257)

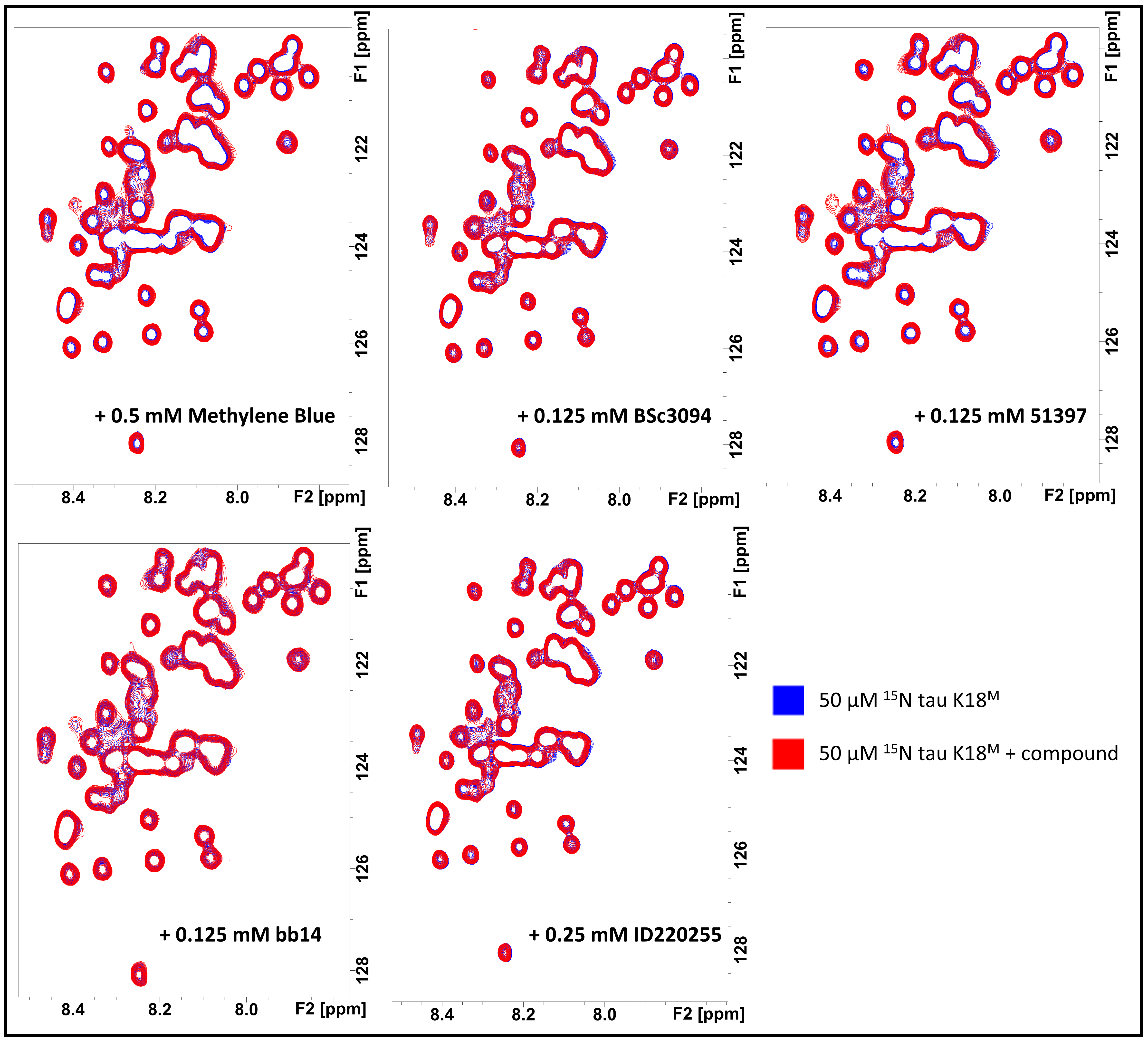

Supplement: Supplementary file 1 [file ijms-21-05257-s001.zip › Figure S1.tif]

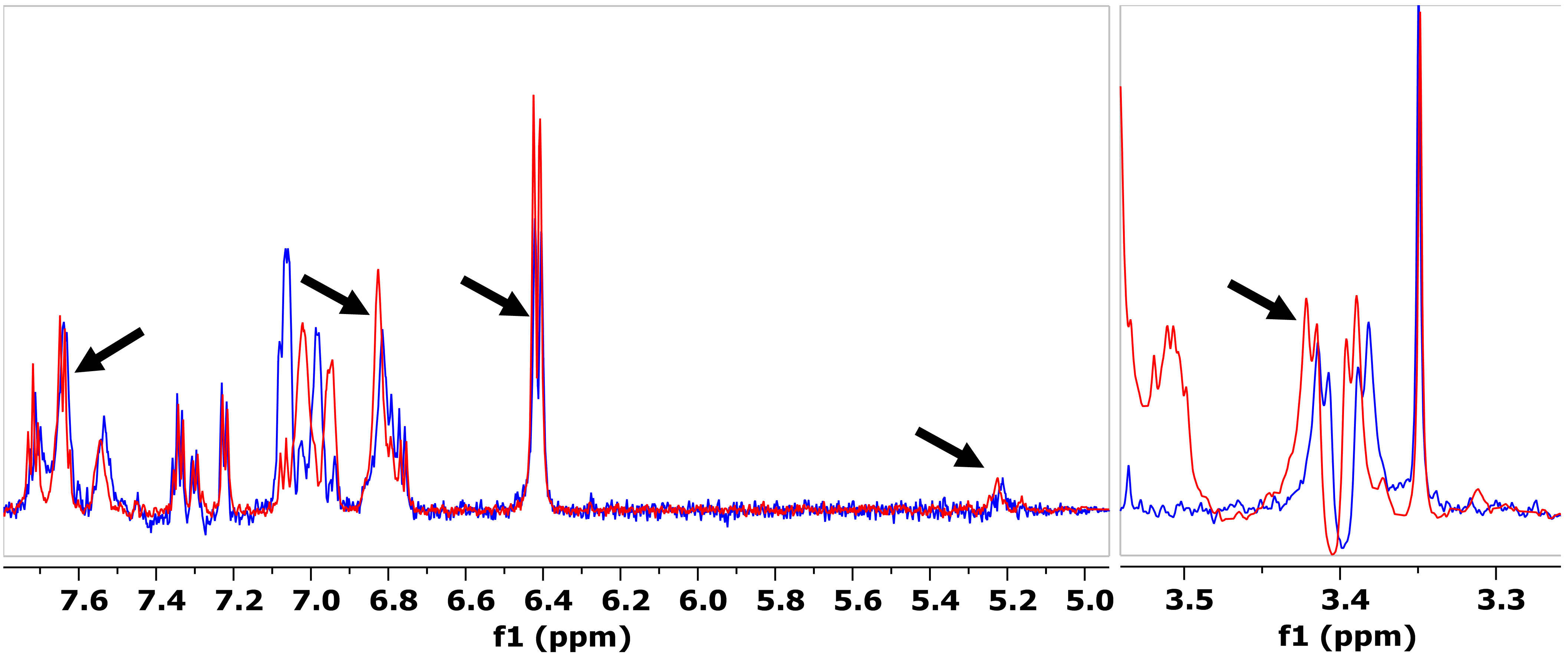

Supplement: Supplementary file 1 [file ijms-21-05257-s001.zip › Figure S10.tif]

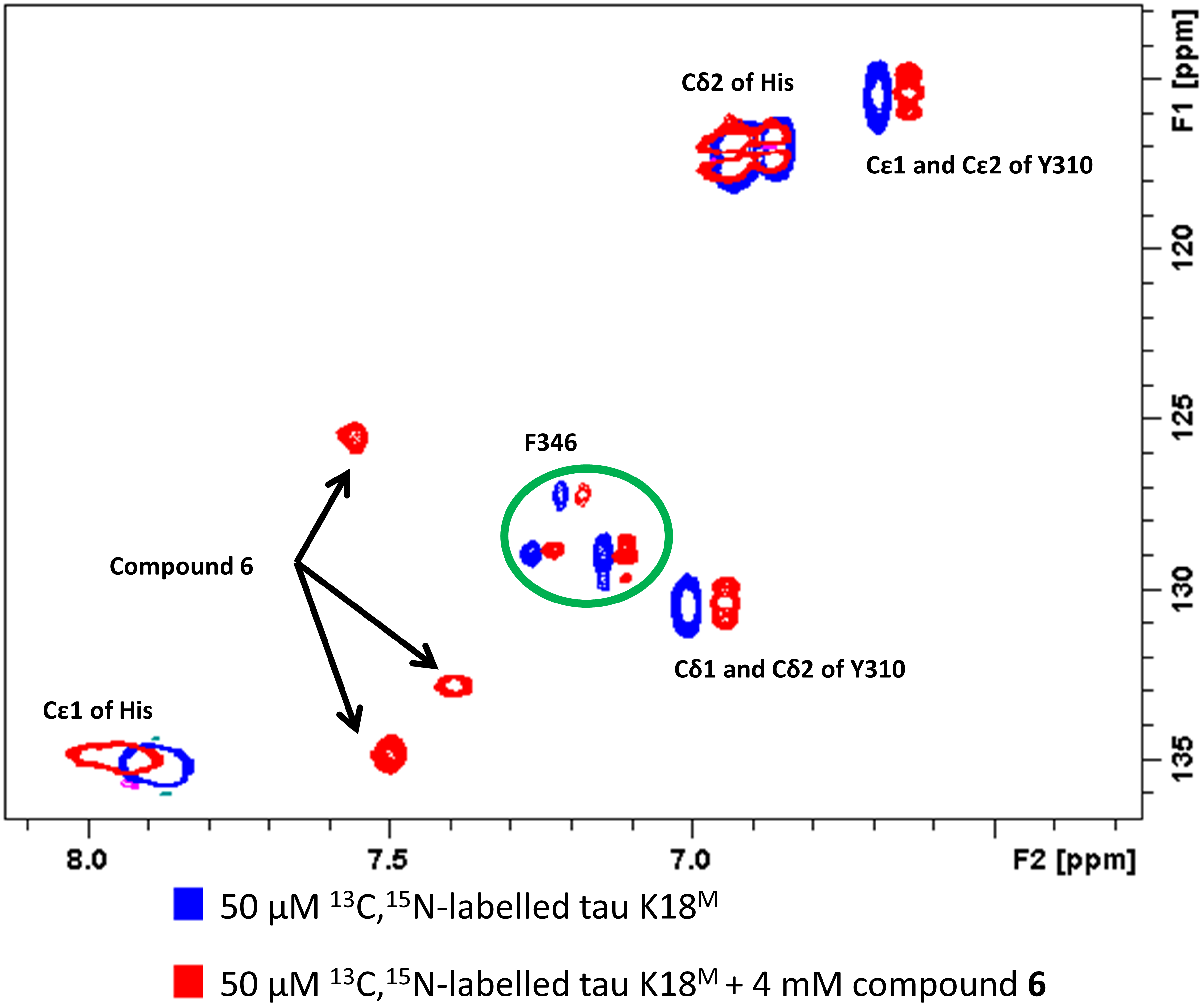

Supplement: Supplementary file 1 [file ijms-21-05257-s001.zip › Figure S2.tif]

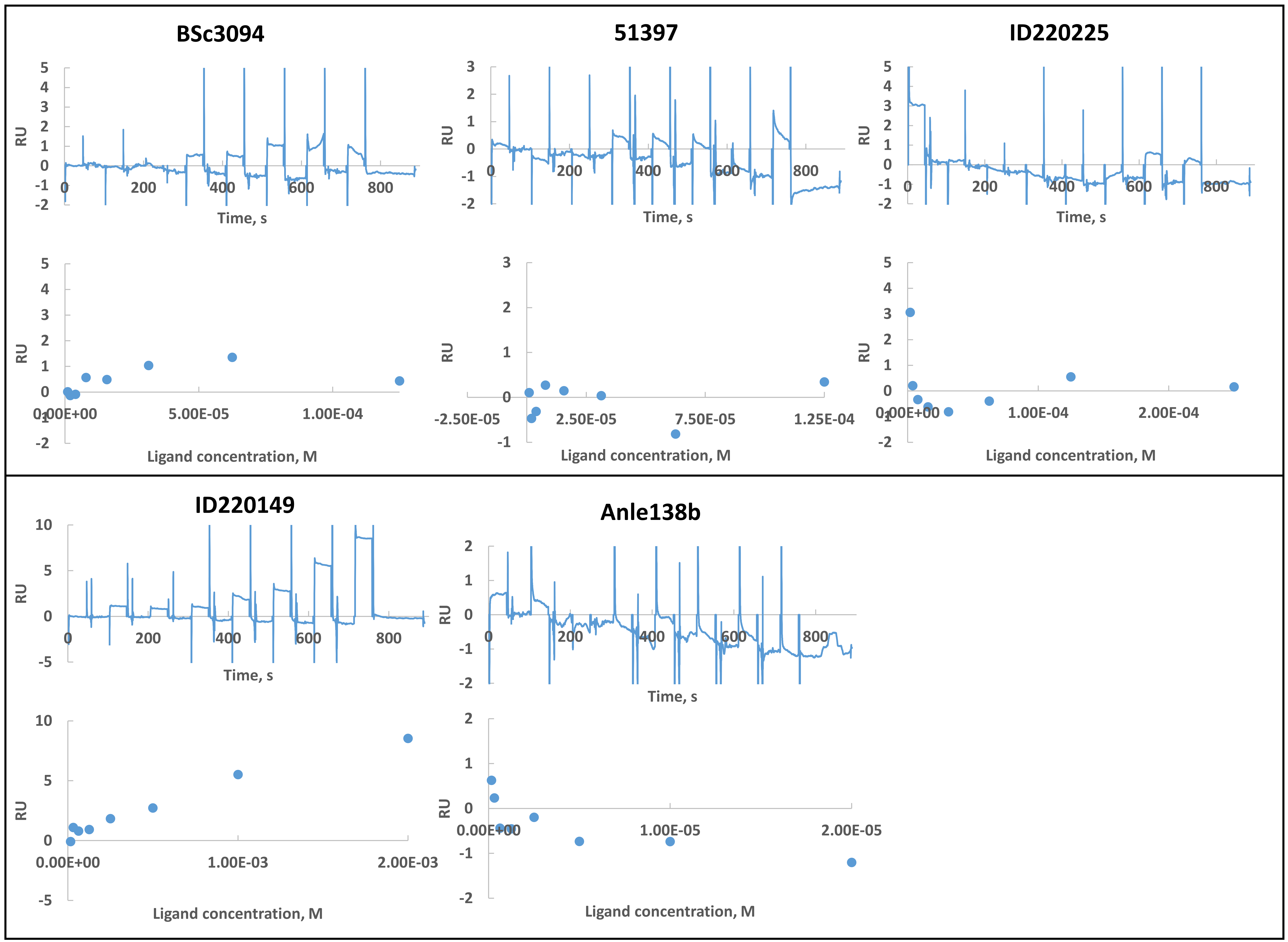

Supplement: Supplementary file 1 [file ijms-21-05257-s001.zip › Figure S3.tif]

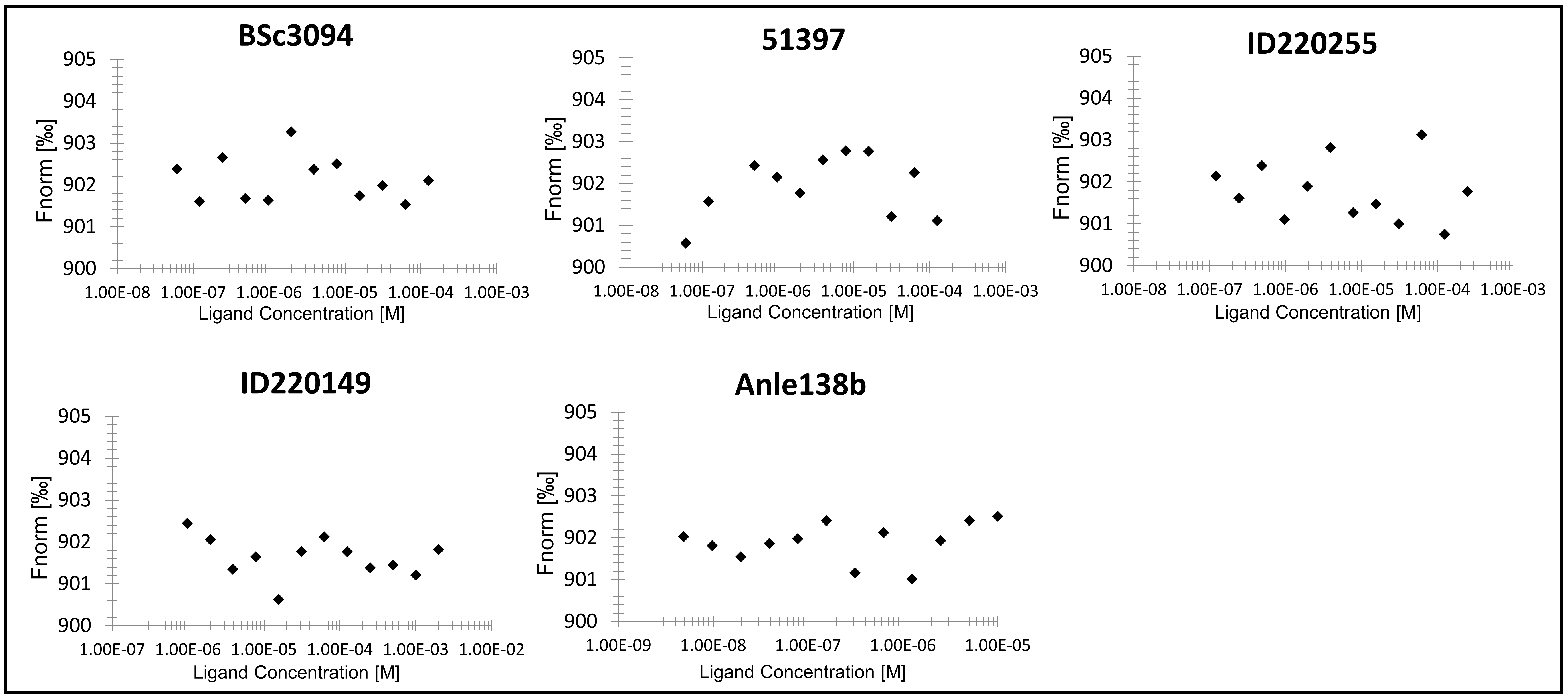

Supplement: Supplementary file 1 [file ijms-21-05257-s001.zip › Figure S4.tif]

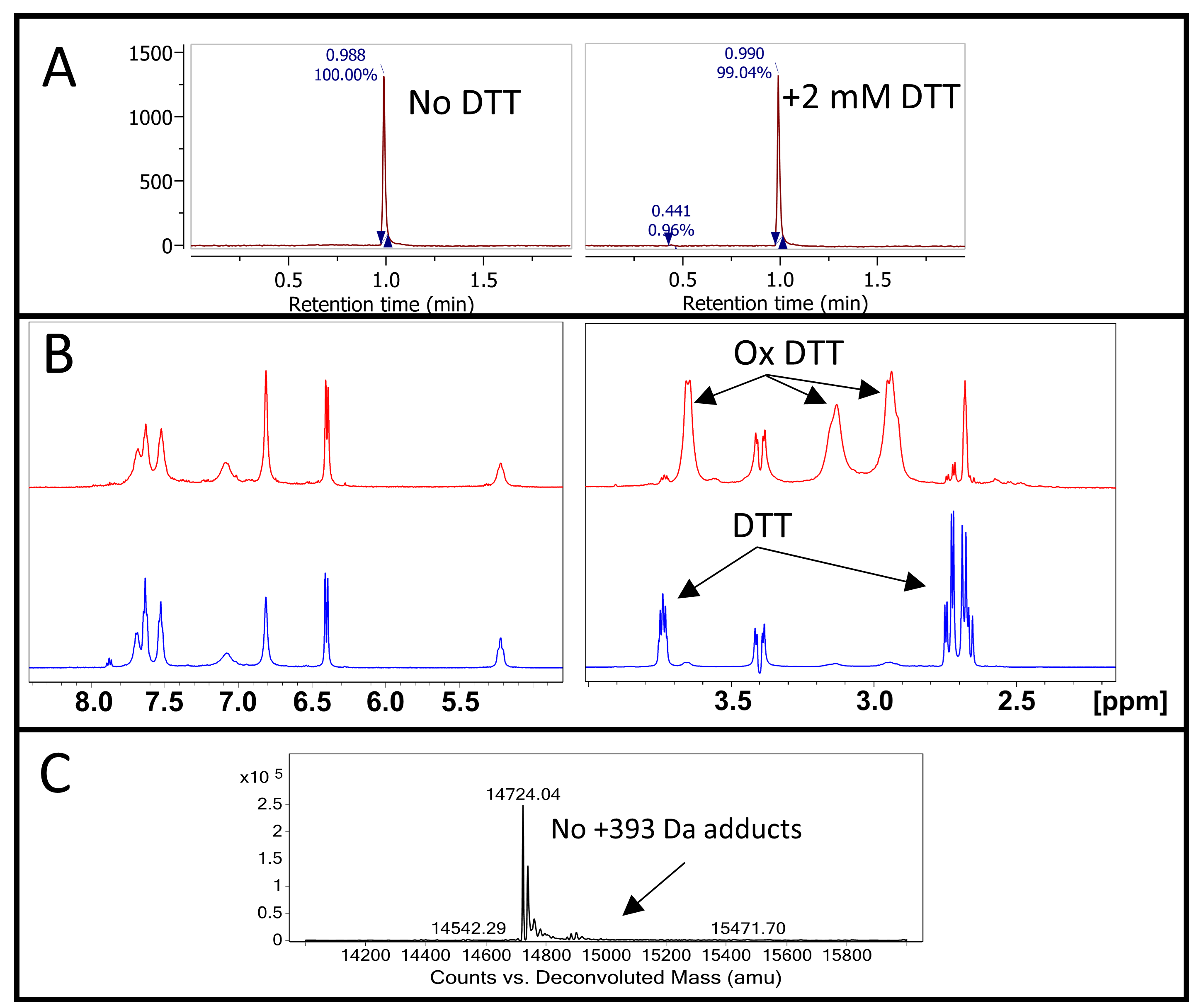

Supplement: Supplementary file 1 [file ijms-21-05257-s001.zip › Figure S5.tif]

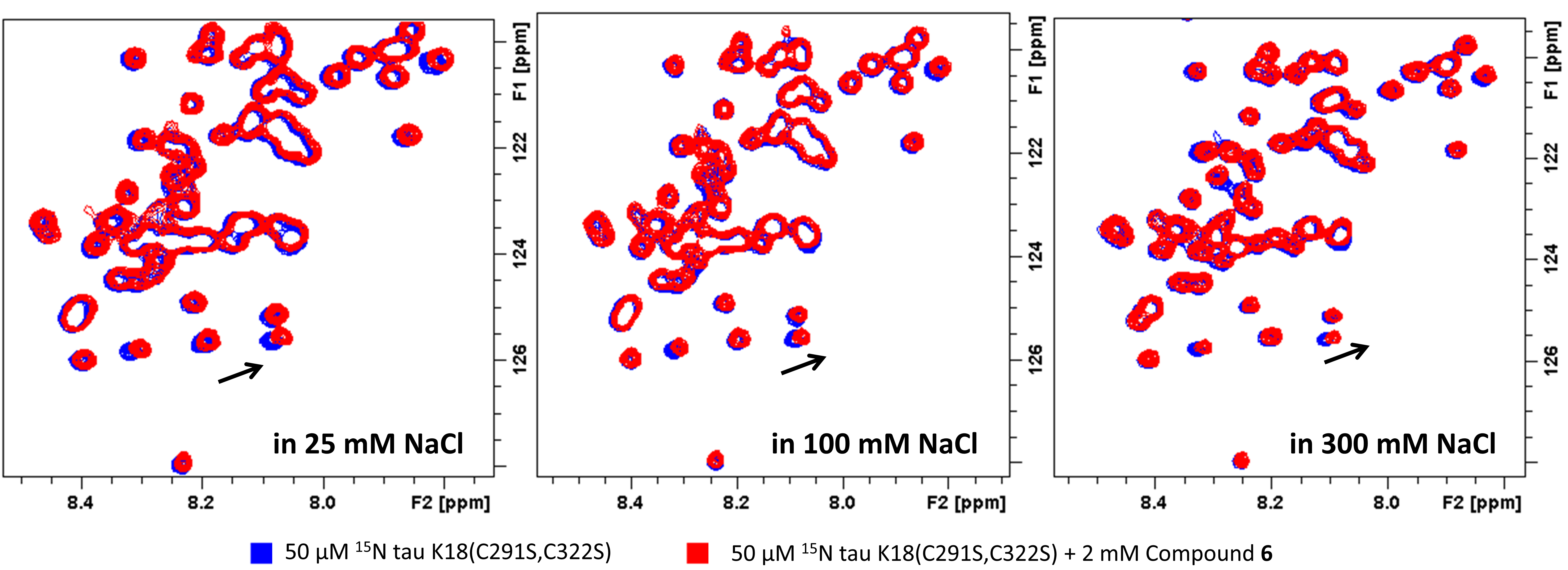

Supplement: Supplementary file 1 [file ijms-21-05257-s001.zip › Figure S6.tif]

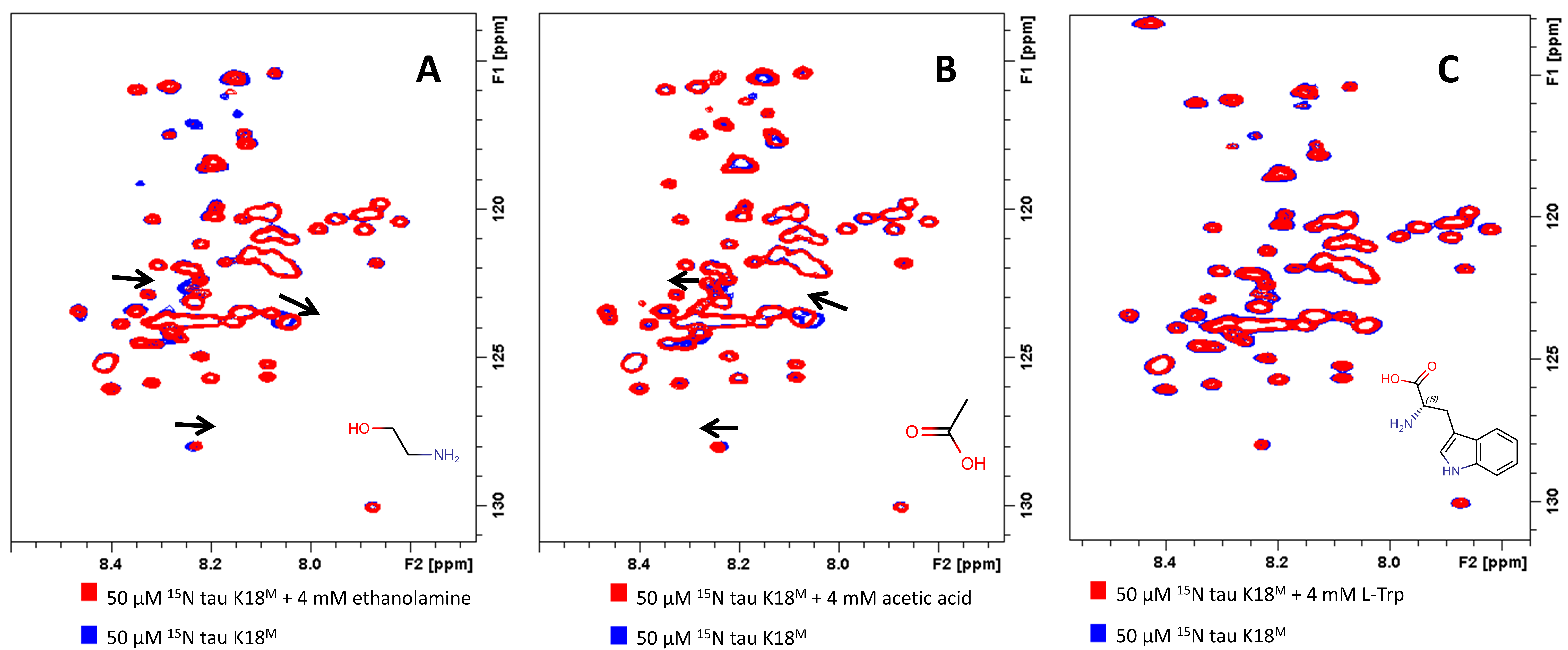

Supplement: Supplementary file 1 [file ijms-21-05257-s001.zip › Figure S7.tif]

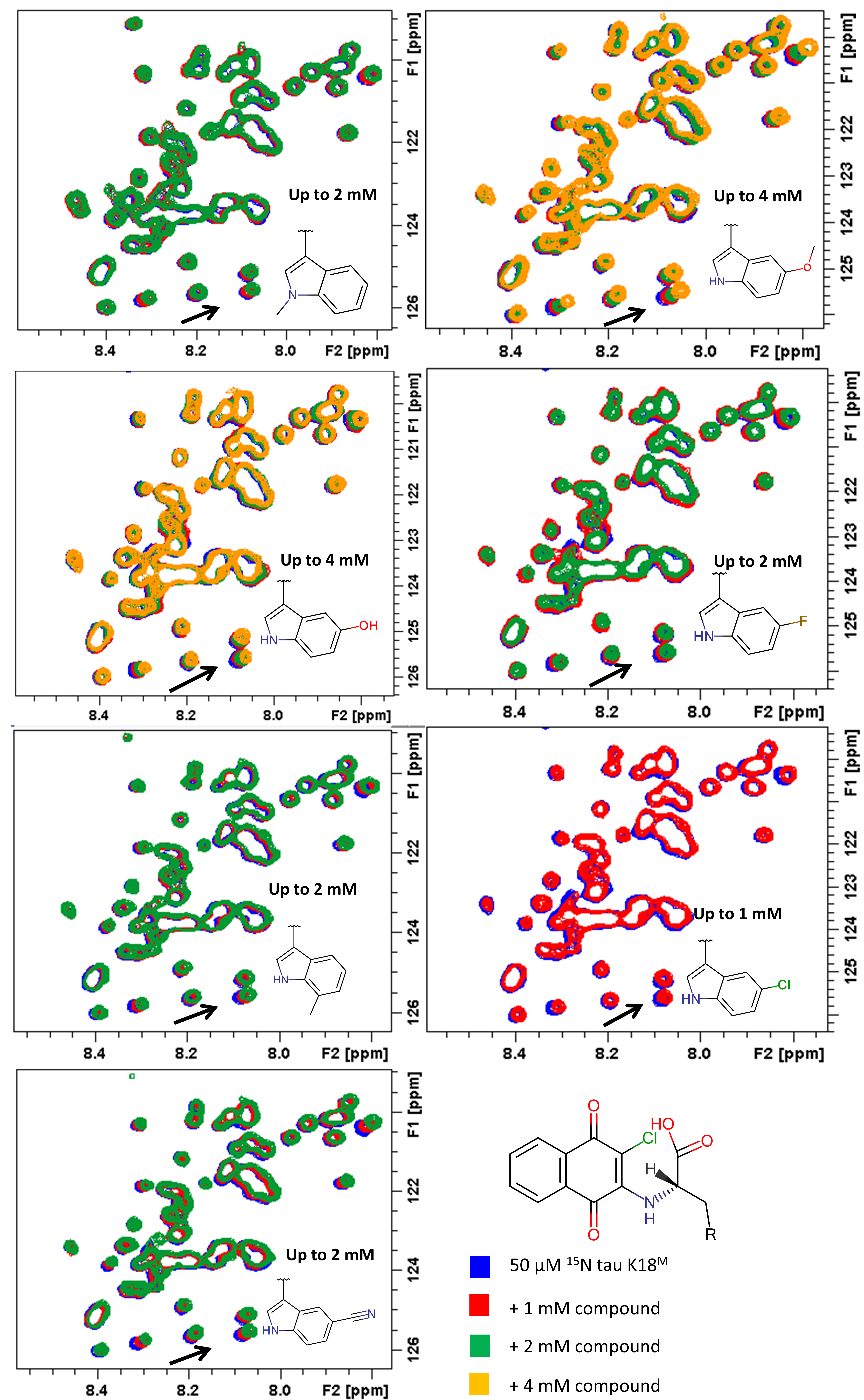

Supplement: Supplementary file 1 [file ijms-21-05257-s001.zip › Figure S8.tif]

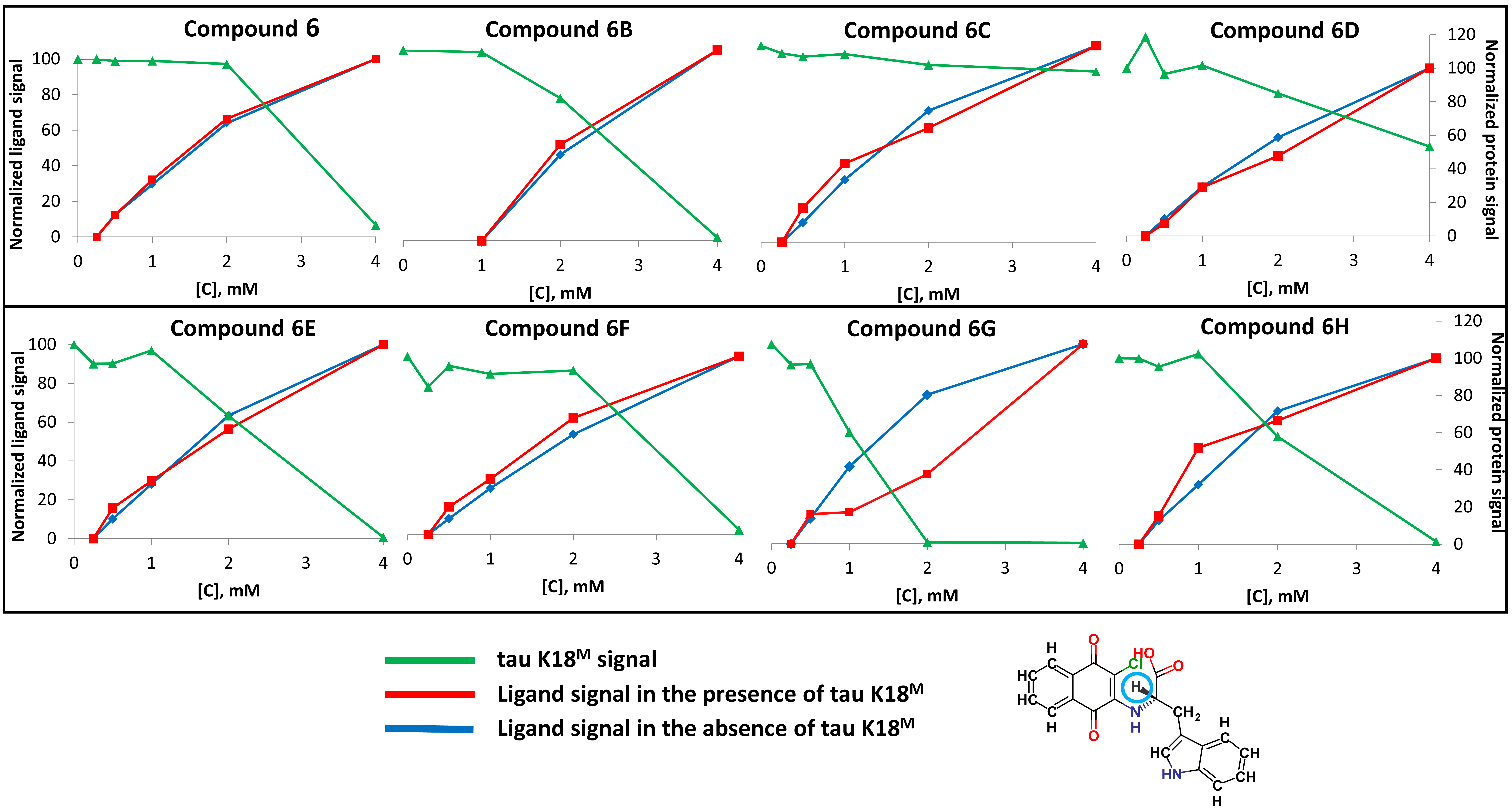

Supplement: Supplementary file 1 [file ijms-21-05257-s001.zip › Figure S9.tif]
